# A chemo-mechanical model for describing sorption hysteresis in a glassy polyurethane

**DOI:** 10.1038/s41598-024-56069-3

**Published:** 2024-03-07

**Authors:** Brandon L. Foley, Sarah M. Matt, Stephen T. Castonguay, Yunwei Sun, Pratanu Roy, Elizabeth A. Glascoe, Hom N. Sharma

**Affiliations:** 1 Lawrence Livermore National Laboratory, 7000 East Avenue, Livermore, CA 94550 USA; 2 Present Address: U.S. Centers for Disease Control and Prevention (CDC), National Institute for Occupational Safety and Health (NIOSH), National Personal Protective Technology Laboratory (NPPTL), 626 Cochrans Mill Road, Pittsburgh, PA 15236 USA

**Keywords:** Glassy polymers, Sorption hysteresis, Polymer relaxation, Berens–Hopfenberg kinetics, Physical chemistry, Thermodynamics, Glasses

## Abstract

Hysteretic sorption and desorption of water is observed from 0 to 95% relative humidity and 298–333 K on a glassy polyurethane foam. It is postulated that sorption-induced swelling of the glassy polyurethane increases the concentration of accessible hydrogen-bonding adsorption sites for water. The accessibility of sites is kinetically controlled due to the restricted thermal motions of chains in the glassy polymer, causing a difference in accessible site concentrations during sorption and desorption. This discrepancy leads to hysteresis in the sorbed concentrations of water. A coupled chemo-mechanical model relating volumetric strain, adsorption site concentration, and sorbed water concentration is employed to describe water sorption hysteresis in the glassy polyurethane. This model not only describes the final mass uptake for each relative humidity step, but also captures the dynamics of water uptake, which exhibit diffusion and relaxation rate-controlled regimes.

## Introduction

A critical hurdle for understanding the equilibrium of sorbate-polymer systems is the dependence on the sorption history of the polymer^1–10^. Many materials exhibit near-perfect overlap of sorption isotherms during the uptaking and outgassing of vapor species^11^. The sorbed concentration of species in these materials is a state function that is path-independent, and temperature and sorbate pressure are sufficient for defining the equilibrium state^11^. However, some materials display more complex behaviors, with a mismatch or hysteresis between the sorption and desorption isotherms. In these materials, the apparent equilibrium is path-dependent, requiring knowledge of prior states of the polymer to sufficiently define the final state of the sorbate-polymer system.

Understanding vapor sorption hysteresis enables better forecasting of material source/sink behaviors in enclosed environments, efficacy as moisture sealants, and properties as separation membranes in a range of humidity conditions^1–10^. In this work, we investigate a glassy polyurethane foam that exhibits sorption hysteresis at all relative humidities. Water sorption in polyurethane materials has been extensively studied^12–21^, but to our knowledge, a thorough discussion on sorption–desorption hysteresis in glassy polyurethanes does not exist. Rubbery polyurethane does not exhibit hysteresis during water sorption-desorption^15,22^, suggesting that the glassy nature of our polyurethane is the origin of the observed hysteresis.

Sorption hysteresis at low relative pressures in glassy polymers is often attributed to changes in the polymer properties caused by sorption-induced swelling. These changes persist during desorption because the restricted thermal motions of polymer chains slows or prevents relaxation to an equilibrium state^3–6,11,23,24^. Consequently, the polymer properties during sorption and desorption differ, resulting in sorption hysteresis^3–6,11,24^. The dynamics of these relaxations often manifest as non-Fickian sorption behavior, and have been observed in polyurethanes previously^25–28^. This non-Fickian behavior is typically captured by exponential decay functions in the form of empirical Berens–Hopfenberg models, which are specific to each humidity step and do not describe sorption hysteresis^25–27^.

**Figure 1:**
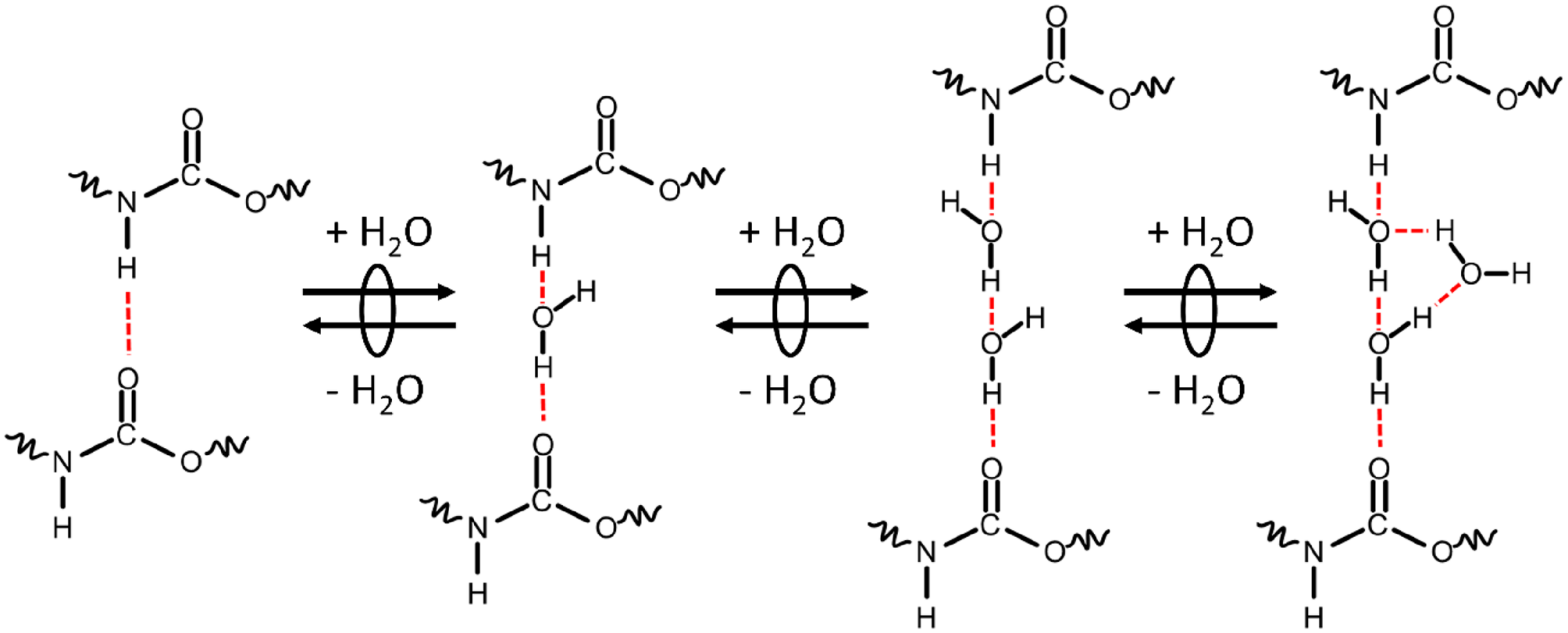
A simplified schematic for multilayer water sorption in polyurethane via hydrogen bonding.

Water sorption on polyurethanes and amide-containing epoxy resins occur primarily by hydrogen bonding at urethane and amide groups, respectively^29,30^. Water sorption increases with urethane group concentration and spectroscopic investigations confirm the sorption of water at amine (–N–H), carbonyl (–C=O), and ether (–C–O–C–) components of the urethane group (shown schematically in Fig. 1)^13,14,16–18,31–34^. Not all urethane groups present in polyurethanes are accessible to water, as assessed by H–D exchange^22,35,36^. Sorbed water concentration is proportional to the concentration of accessible urethane groups, which changes as a function of the sample history^22,35,36^. An archetypal example of this type of phenomenon is water sorption on glassy amorphous cellulose. Water sorbs by hydrogen bonding with hydroxyl groups in amorphous cellulose and also exhibits sorption hysteresis^5,6,31,32,37,38^. It has been demonstrated that hysteresis is likely caused by changing accessibility of hydrogen bonding sites with hygroscopic swelling of the material^5,6,31,32,37,38^. As the material swells, more sites become available, and some of these sites are retained during desorption until the material is completely dried due to the slow relaxation of the glassy polymer chains^5,6^.

In this work, we demonstrate that increasing concentrations of accessible hydrogen bonding sites due to sorption-induced swelling provides a plausible explanation for the observed water sorption–desorption hysteresis on glassy polyurethane. We develop a coupled chemo-mechanical model based on the assumption that multilayer sorption in the polyurethane is described by the Guggenheim–Anderson-de Boer (GAB) model^39–41^ with site densities that are functions of the volumetric strain. This model captures the non-Fickian water sorption dynamics with a kinetic function that intertwines the observed sorption hysteresis to the polymer relaxation dynamics.

## Results and discussion

### Dynamic vapor sorption

**Figure 2:**
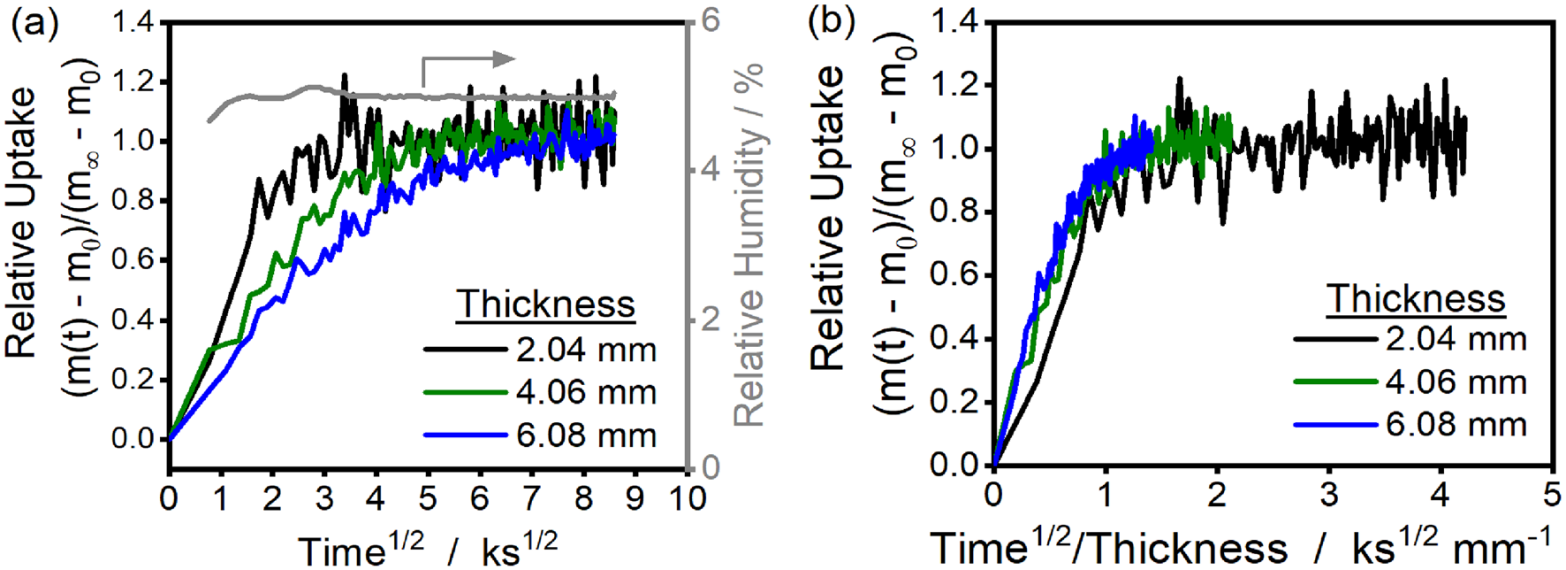
(**a**) Relative uptake versus time^1/2^ for samples of varying thickness (2–6 mm) at 323 K for steps in relative humidity from 0 to 5%. The gray line is the relative humidity (right y-axis). (**b**) Relative uptake versus time^1/2^ divided by sample thickness for samples of varying thickness (2–6 mm) at 323 K for steps in relative humidity from 0 to 5%. Sample width and length dimensions are ~ 15 mm × 20 mm.

The dynamic uptake of water when step-changing the relative humidity from 0 to 5% at 323 K is reported in Fig. 2a for 2-, 4-, and 6-mm thick samples with heights and widths of ~ 15 ×  ~ 20 mm. Increasing sample thickness causes slower relative uptake rates at this humidity condition. A test for evaluating whether the limiting process for vapor uptake is relaxation of polymer chains or Fickian diffusion is to determine if relative uptake curves overlay when plotted against time^1/2^ or time^1/2^/thickness, respectively^42^. In Fig. 2b, uptake curves overlay as a function of time^1/2^/thickness, demonstrating that water sorption is limited by Fickian diffusion for the 0–5% relative humidity (RH) step.

**Figure 3:**
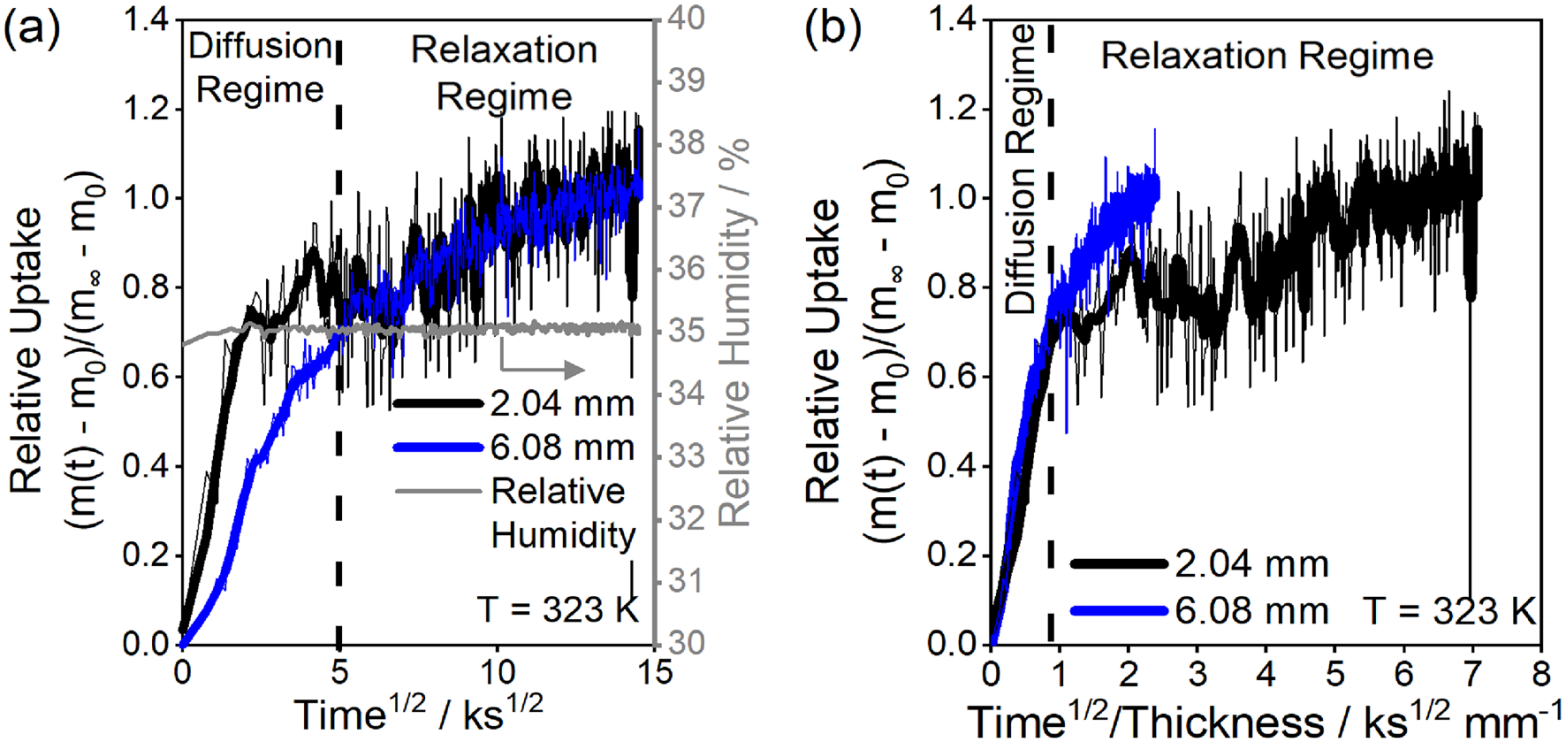
Comparison of the relative uptake for the 2.04 mm and 6.08 mm thick glassy polyurethane samples for the 35% humidity step as a function of (**a**) time^1/2^ and (**b**) time^1/2^/thickness. The uptake curves overlay at short times in (**b**) when compared as a function of time^1/2^/thickness, indicating diffusion-controlled uptake from time^1/2^/thickness ~ 0–1 ks^1/2^ mm^−1^. The uptake curves overlay at long times in (**a**), indicating a relaxation-controlled uptake regime from time^1/2^/thickness > 1 ks^1/2^ mm^−1^. Thin lines are raw experimental data, thick lines are ~ 2.4 ks moving averages. The gray line in (**a**) is the relative humidity (secondary y-axis).

The dynamics for the uptake curve when step-changing the relative humidity from 30 to 35% are shown in Fig. 3a, where again the 2 mm-thick glassy polyurethane has faster relative uptake than the 6 mm-thick sample. Instead of plateauing at longer times, as in Fig. 2, uptake continues to increase slowly for the 35% RH step in Fig. 3a. At short times (< ~ 25 ks), the uptake curves overlay when compared as a function of time^1/2^/thickness (Fig. 3b), but at longer times (> ~ 25 ks) the uptake curves overlay only when compared as a function of time (Fig. 3a). In the region where uptake scales with time^1/2^/thickness, the rate of water sorption is limited by Fickian diffusion. At longer equilibration times, water is in diffusive quasi-equilibrium and the sorption rate is limited by the kinetics of polymer chain relaxations.

**Figure 4:**
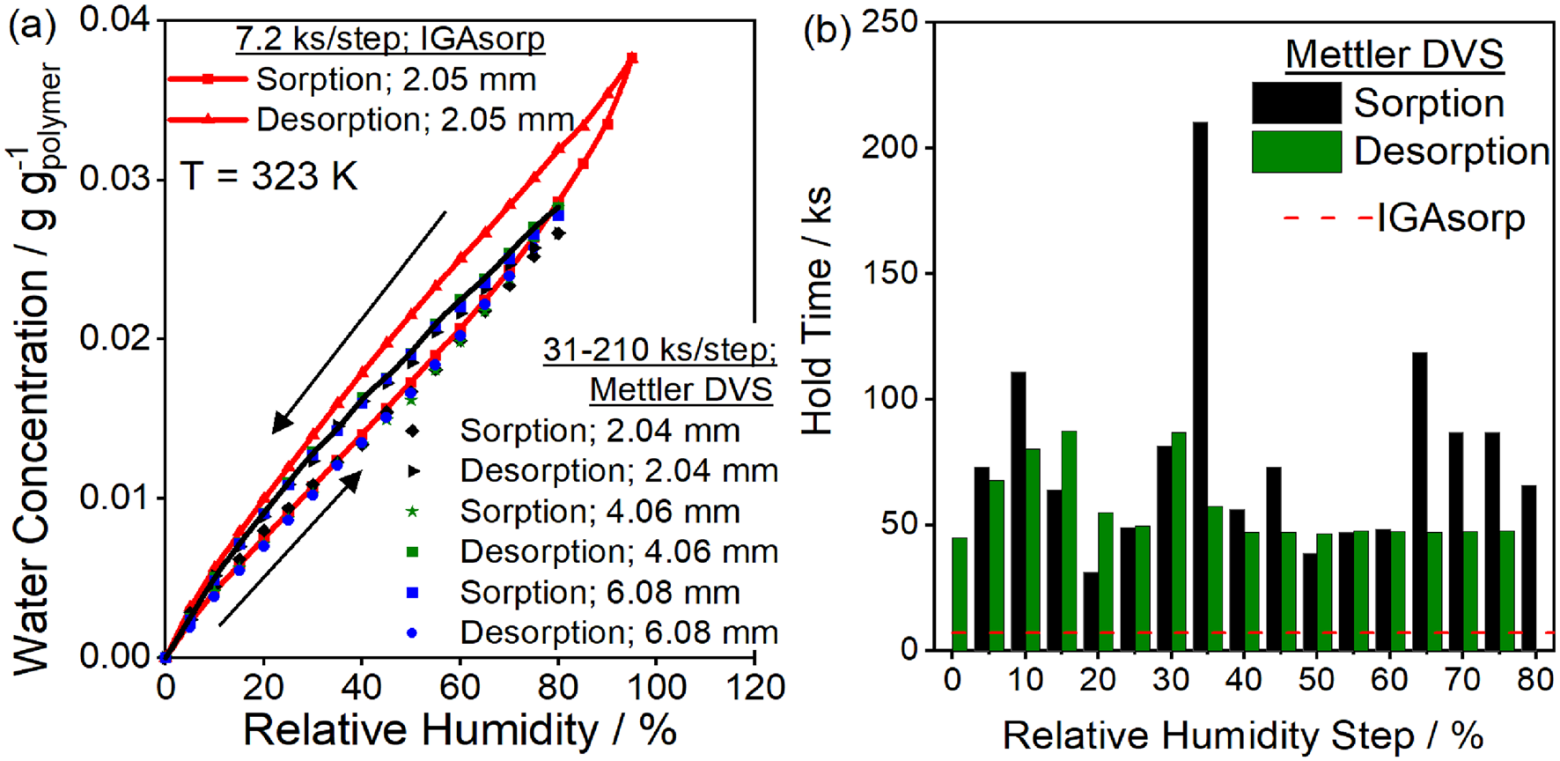
(**a**) Sorbed water concentration versus relative humidity at 323 K for samples of varying thickness on the Hiden Isochema IGAsorp and Mettler DVS. Time per humidity step was a constant 7.2 ks per humidity step on the IGAsorp and varied between 31 and 210 ks per humidity step on the Mettler DVS. The exact length of time for each humidity step is shown in (**b**). The maximum humidity step was 95% RH for the IGAsorp and 80% RH for the Mettler DVS. Sorption isotherms overlayed for all samples, desorption isotherms were lower for samples exposed to a maximum of 80% RH compared to the sample exposed to 95% RH but did not vary with thickness. Sorption–desorption hysteresis is observed for all samples, instruments, and equilibration times.

In Fig. 3, the mass uptake has a slight positive slope even after 200 ks, suggesting that the hold time for the humidity step may alter the final mass uptake. To investigate this effect, water sorption was measured on a 2.05 mm thick sample from 0 to 95% RH with 7.2 ks hold times for each humidity step. This result is compared to the water sorption with 31–210 ks hold times on samples of thickness 2.04, 4.06, and 6.08 mm from 0 to 80% RH. The mass at the end of each sorption and desorption step is reported in Fig. 4a, and the hold times for each step are reported in Fig. 4b. The sorption isotherm is the same for each sample, regardless of thickness, hold time, or instrument. Thus, increasing hold times of the experiment does not appreciably change the resulting sorption uptake, suggesting the polymer chain relaxation rates are too slow to reach equilibrium on laboratory time scales. Each sample exhibited hysteresis with the desorption isotherms giving higher mass uptake than the sorption isotherms. The desorption isotherms from 80% RH were the same for all three samples of varying thickness but had lower mass uptakes than the sample exposed to a 95% RH maximum.

**Figure 5:**
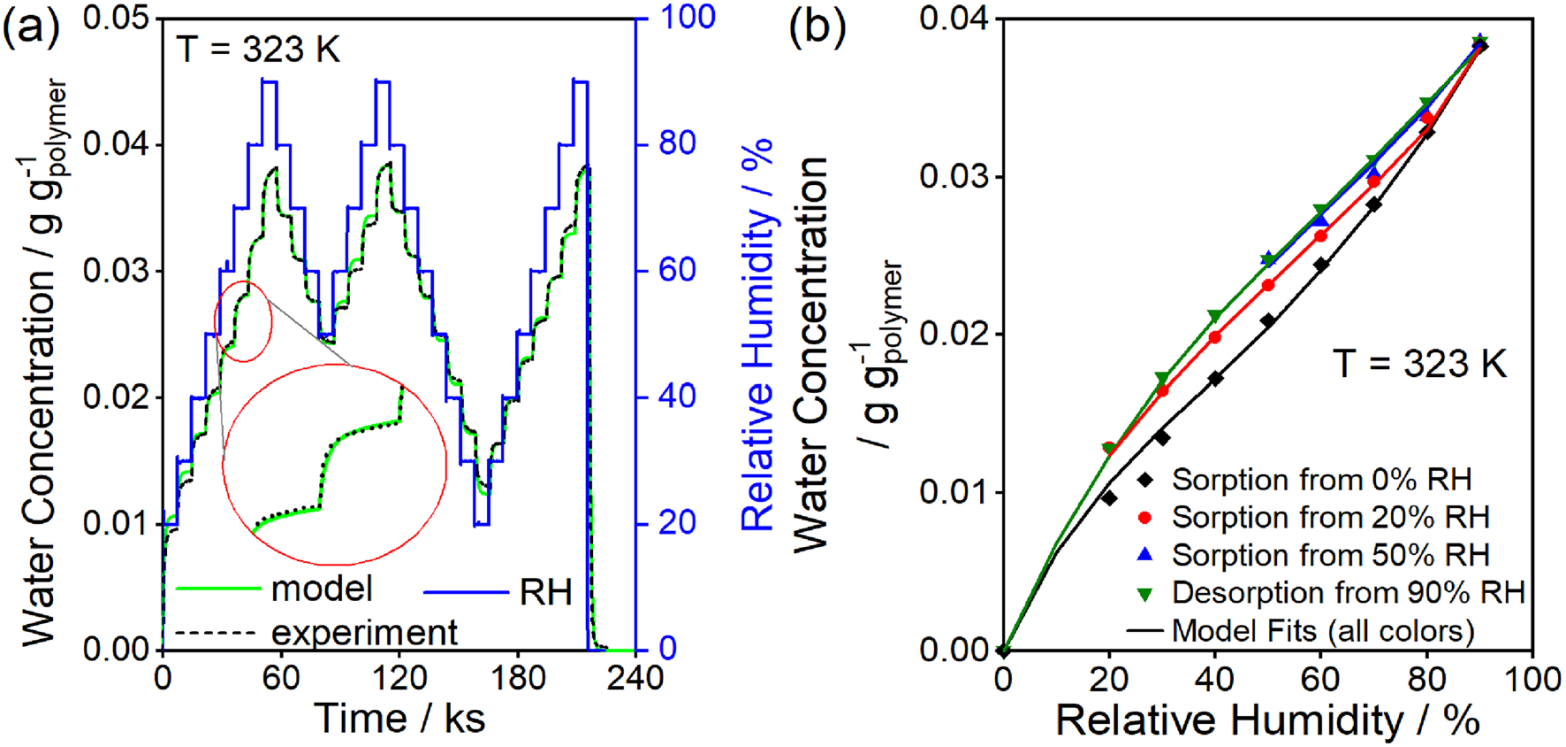
(**a**) Dynamic uptake curves for 15 $\times$ 20 $\times$ 0.48 mm slab glassy polyurethane sample for 7.2 ks equilibration times for each humidity step. Inset: an enlarged view of the dynamic uptake during the 60% RH step to highlight the still increasing mass at long times and the match between model and experiment. (**b**) The final uptake for each RH step from (**a**) during the initial sorption (black filled diamond), desorption from 90% RH (green filled inverted triangle), and sorption from 50% (blue filled triangle) and 20% (red filled diamond) RH. Each sorption curve depends on the minimum RH reached before increasing the humidity. Solid lines are full dynamic model predictions.

In Fig. 4, the desorption isotherms depend on the maximum relative humidity step. In Fig. 5, the effect of varying the minimum relative humidity on the sorption–desorption dynamics (Fig. 5a) and isotherm hysteresis (Fig. 5b) is investigated on a 0.48 mm thick polyurethane sample. The inset in Fig. 5a shows that initially the rate of uptake is fast for a given humidity step, but the mass continues increasing slowly due to polymer relaxations even after 7.2 ks, like the uptake curve shown in Fig. 3. In Fig. 5b, the sorption isotherms differed depending on whether the sorption isotherm started from 0%, 20%, or 50% RH.

**Figure 6:**
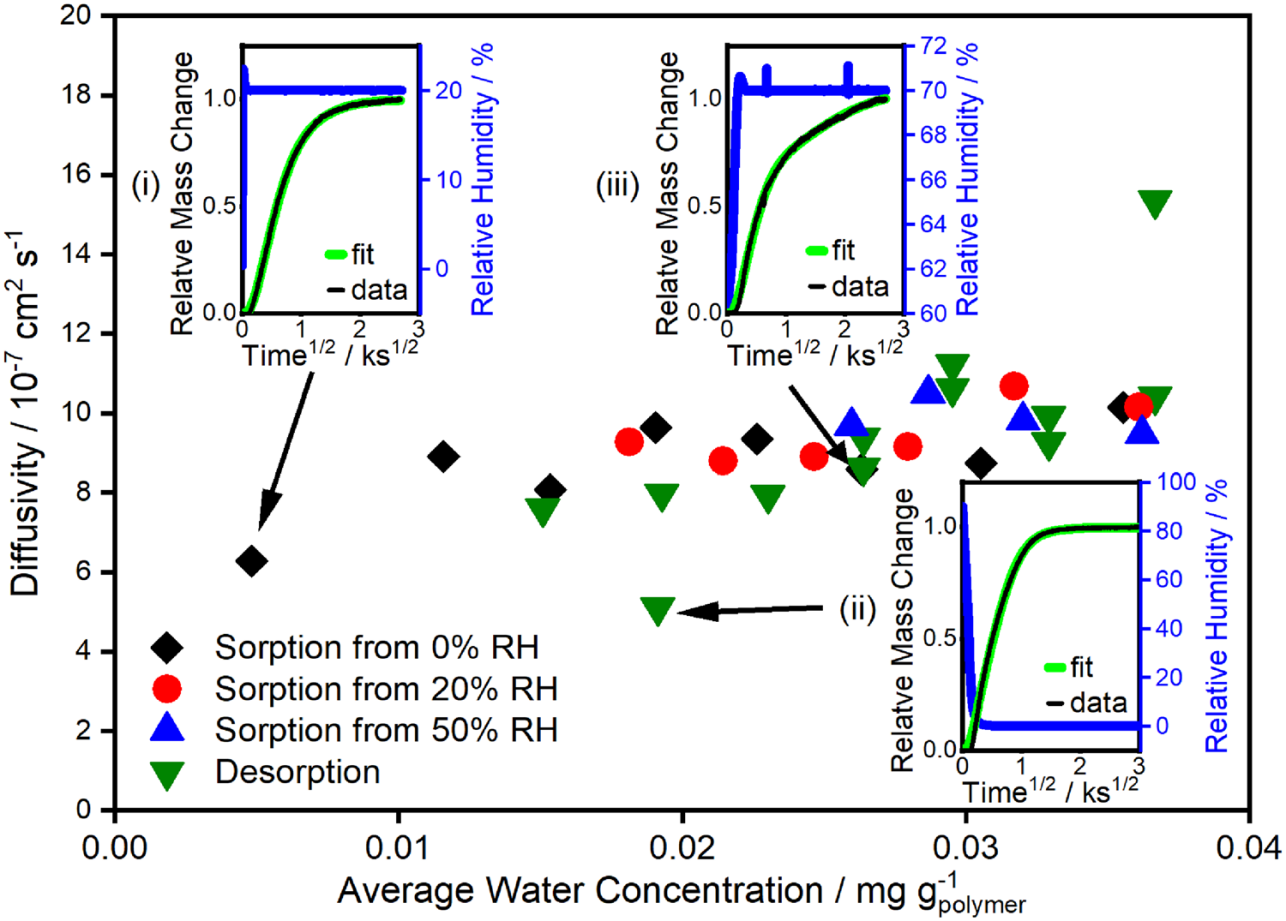
Diffusivity as a function of sorbed water concentration for sorption and desorption steps as estimated by fitting Fick’s second law to dynamic sorption at 323 K for the 0.48 mm polyurethane sample (Fig. 5). Data points are located at the average of the initial and final concentration for each humidity step. Insets show fits (green) to experimental data (black) for the (i) 0 to 20% RH step, (ii) 90 to 0% RH step, and the (iii) 60 to 70% RH step. Relative humidity (blue) is shown on the right y-axis.

Water sorption in the polyurethane was found to be primarily diffusion limited, as determined by the analysis in Figs. 2 and 3. To assess whether the diffusivity changes as a function of sorbed concentration, we fit an analytical solution^43^ (see “Estimating diffusivity for each humidity step in Fig. 6” section) of Fick’s second law with a Berens–Hopfenberg^8^ kinetic term to each of the dynamic sorption and desorption steps shown in Fig. 5. The Berens–Hopfenberg term here empirically describes non-Fickian dynamics at long timescales to obtain a first approximation of diffusivity and its dependence on sorbed concentration. This term will be replaced later with a constitutive model that simultaneously captures the non-Fickian dynamics and the hysteresis observed over the entire isotherm. The diffusivity estimates from these fits are reported as a function of mean sorbed water concentration in Fig. 6.

The initial 0 to 20% humidity step (Fig. 6, inset (i)) and 90 to 0% humidity steps (inset (iii)) have dynamics that are almost entirely Fickian. In contrast, the sorption dynamics for the 60 to 70% humidity step (inset (ii)) exhibits strong non-Fickian behavior after ~ 1 ks, further demonstrating the findings of Fig. 3 that bulk relaxation phenomena contribute to the sorption dynamics. Water sorption within glassy polyurethanes could lead to plasticization by disrupting interchain hydrogen bonding which, in turn, increases chain mobility and alters material mechanical properties^18^. Materials that exhibit plasticization, such as polybenzimidazoles, have concentration dependent diffusivities that change by several orders of magnitude^25^. In contrast, the diffusivity of water in our polyurethane appears relatively invariant with water concentration, suggesting plasticization is not significantly altering the diffusivity of water in our polyurethane.

### Derivation and fitting of the swelling GAB model

Water sorption isotherms on glassy polyurethane exhibit sorption hysteresis (Figs. 4, 5) with dynamics that are initially diffusion controlled but transition to polymer relaxation rate-controlled regimes at longer time scales (Figs. 2, 3). These observations suggest that the apparent hysteresis is caused by non-equilibrium relaxation of the glassy polymer chains^3–6,11^.

Herein, we describe a model that simultaneously captures the sorption dynamics with both fast diffusion and slow relaxation regimes and the observed sorption–desorption hysteresis. Discussions on other hysteresis models, including the poromechanics model derived by Chen et al.^5^, the non-equilibrium lattice fluid model^2,4^, and the Vrentas and Vrentas^3^ model, are provided in Sect. S1 of the Supporting Information. For the model developed herein, we consider the following properties:

(i) Multiple water molecules sorb preferentially at discrete polyurethane sorption sites via hydrogen bonding, and other sorption modes are negligible^22,29,30,35,36^.
(ii) Transport of water into the polyurethane occurs via Fickian diffusion (Figs. 2, 3)
(iii) The accessibility of hydrogen bonding sites changes with the volumetric strain^5,6,22,35,36^.
(iv) The change in accessible sites is kinetically-controlled by the relaxation of the glassy polymer chains (Fig. 3) ^23,25–27^.

These assumptions lead to the diffusion transport equation (Eq. (1))^44^:

1 $$\frac{\partial c}{\partial t}=\frac{\partial }{\partial x}\frac{Dc}{RT} \frac{\partial \mu }{\partial x},$$

where $c$ is the concentration of sorbed water, $D$ is the diffusion coefficient, $x$ is the depth into the thin slab, $t$ is time, $R$ is the gas constant, $T$ is temperature, and $\mu$ is chemical potential. Often the right-hand side of Eq. (1) is written as $D\frac{{\partial }^{2}c}{\partial {x}^{2}}$ which requires ideal sorption in the polymer and constant diffusivity. The concentration is written as a product of the local average number of sorbed water molecules per site, $\overline{\theta }\left(x,t\right)$, and the site concentration, $L\left(x,t\right)$, which can both depend on position and time. Substitution of this into Eq. (1) gives Eq. (2).

2 $$\frac{\partial \overline{\theta }L}{\partial t}=\frac{\partial }{\partial x}\frac{D\overline{\theta }L}{RT} \frac{\partial \mu }{\partial x}.$$

The chemical potential for species sorbed at discrete sites is given as Eq. (3):

3 $$\mu ={\mu }^{{\text{o}}}+RT{\text{ln}}(\overline{\theta }\text{/}{\overline{\theta }}^{{\text{o}}}),$$

where the reference state is taken as ${\overline{\theta }}^{{\text{o}}}=1$. Substitution of Eq. (3) into Eq. (2) gives Eq. (4):

4 $$\frac{\partial \overline{\theta }L}{\partial t}=\frac{\partial }{\partial x}\frac{D\overline{\theta }L}{RT} \frac{\partial \mathit{RT} {\ln\overline{\theta }}}{\partial x}.$$

Applying the chain rule on the derivatives in Eq. (4) and simplifying gives Eq. (5):

5 $$L\frac{\partial \overline{\theta }}{\partial t}+\overline{\theta }\frac{\partial L}{\partial t}=\frac{\partial }{\partial x}DL\frac{\partial \overline{\theta }}{\partial x}.$$

The boundary conditions for this partial differential equation are (Eq. 6):

6 $$\begin{gathered} \overline{\theta }\left( {x = 0,t} \right) = \overline{\theta }_{{{\text{eq}}}} \left( {a_{{\text{w}}} \left( t \right)} \right) \hfill \\ \left. {\frac{{\partial \overline{\theta }}}{\partial x}} \right|_{{x = \frac{h}{2}}} = 0, \hfill \\ \end{gathered}$$

where ${\overline{\theta }}_{{\text{eq}}}$ is the equilibrium sorbed water per site at the gas-phase water activity ${a}_{{\text{w}}}$ and $h$ is the sample thickness. The initial condition for the dried material is $\overline{\theta }\left(x,t=0\right)=0$ and $L(x,t=0)={L}_{i}$.

The equilibrium sorption, ${\overline{\theta }}_{{\text{eq}}}$, is described by the GAB model^39–41^ (Eq. 7):

7 $${\overline{\theta }}_{{\text{eq}}}\left({a}_{{\text{w}}}\right)=\frac{b{a}_{{\text{w}}}}{\left(1-\omega {a}_{{\text{w}}}+b{a}_{{\text{w}}}\right)\left(1-\omega {a}_{{\text{w}}}\right)},$$

where $b$ is the equilibrium constant for the sorption of the first water molecule at a site, $\omega$ is the equilibrium constant for each subsequent water molecule, and ${a}_{{\text{w}}}$ is the activity of water. In this work, we specify the reference pressure of water as the saturation pressure, ${P}_{{\text{w}}}^{{\text{sat}}}(T)$, such that ${a}_{{\text{w}}}={P}_{{\text{w}}}/{P}_{{\text{w}}}^{{\text{sat}}}$ is the relative humidity. This model assumes that monolayer and multilayer equilibrium constants differ because the sorption of the first molecule interacts only with the polymer, but each subsequent water molecule interacts with the polymer and the other water molecules that are already adsorbed (see Fig. 1).

Finally, we require one more equation to have a completely defined system. This missing equation is one describing how site concentrations, $L$, change in time. The change in $L$ is empirically captured by an nth order power-law model (Eq. (8)):

8 $$\frac{\partial L}{\partial t}=\left\{\begin{array}{c}\begin{array}{cc} k\times {\left|{L}_{\infty }-L\left(x,t\right)\right|}^{n}& \forall\; {L}_{\infty }>L\end{array}\\ \begin{array}{cc}-k\times {\left|{L}_{\infty }-L\left(x,t\right)\right|}^{n}& \forall\; {L}_{\infty }<L\end{array}\end{array}\right.,$$

where ${L}_{\infty }$ is the concentration of sites after an infinite relaxation time at a fixed condition, $k$ is a rate constant, and $n$ is the reaction order. In this equation, the driving force is the magnitude of the difference between ${L}_{\infty }$ and the current site concentration. The sign of the function changes to ensure $L$ grows or decays towards ${L}_{\infty }$. Based on the analysis by Chen et al.^5,6^, the equilibrium concentration of accessible adsorption sites is a function of the volumetric strain (${\varepsilon }_{{\text{v}}}$). As a first-order approximation, this relation becomes (Eq. (9)):

9 $${L}_{\infty }={L}_{0}\left({T}_{{\text{ref}}}\right)+\gamma {\varepsilon }_{{\text{v}}},$$

where $\gamma$ is ${\text{d}}{L}_{\infty }\text{/}{\text{d}}{\varepsilon }_{{\text{v}}}$ at small volumetric strains and ${L}_{0}\left({T}_{{\text{ref}}}\right)$ is the equilibrium concentration of sites at ${T}_{{\text{ref}}}$ = 298 K and 0% RH. The volumetric strain is a function of temperature and water concentration, and thus (Eq. 10):

10 $${L}_{\infty }\left(x,t\right)={L}_{0}\left({T}_{{\text{ref}}}\right)+\gamma \left(\alpha \left(T-{T}_{{\text{ref}}}\right)+\beta c\left(x,t\right)\right)={L}_{0}\left(T\right)+\gamma \beta c\left(x,t\right),$$

where ${L}_{0}\left(T\right)={L}_{0}\left({T}_{{\text{ref}}}\right)+\gamma \alpha \left(T-{T}_{{\text{ref}}}\right)$ is the equilibrium site concentration at 0% RH and temperature $T$, $c(x,t)=\overline{\theta }(x,t)L(x,t)$ is the concentration of sorbed water, and $\alpha$ and $\beta$ are the thermal and hygroscopic expansion coefficients. Substitution of Eq. (10) into Eq. (8) gives Eq. (11):

11 $$\frac{\partial L(x,t)}{\partial t}=\left\{\begin{array}{c}\begin{array}{cc} k\times {\left|{L}_{0}\left(T\right)+\left(\gamma \beta \overline{\theta }\left(x,t\right)-1\right)L(x,t)\right|}^{n}& \forall \;{L}_{\infty }>L\end{array}\\ \begin{array}{cc}-k\times {\left|{L}_{0}\left(T\right)+\left(\gamma \beta \overline{\theta }\left(x,t\right)-1\right)L(x,t)\right|}^{n}& \forall \;{L}_{\infty }<L\end{array}\end{array}\right.,$$

where the time-derivative of $L$ is now only a function of $\overline{\theta }$ and $L$. Equation (11) is a substitute for the Berens–Hopfenberg model^8^ for describing polymer relaxations, with the advantage that it models the relaxation dynamics for all humidity steps simultaneously, uses polymer state variables to describe the dynamics, and captures the sorption hysteresis, as demonstrated in the following section.

### Fitting the swelling GAB model to experimental data

**Figure 7:**
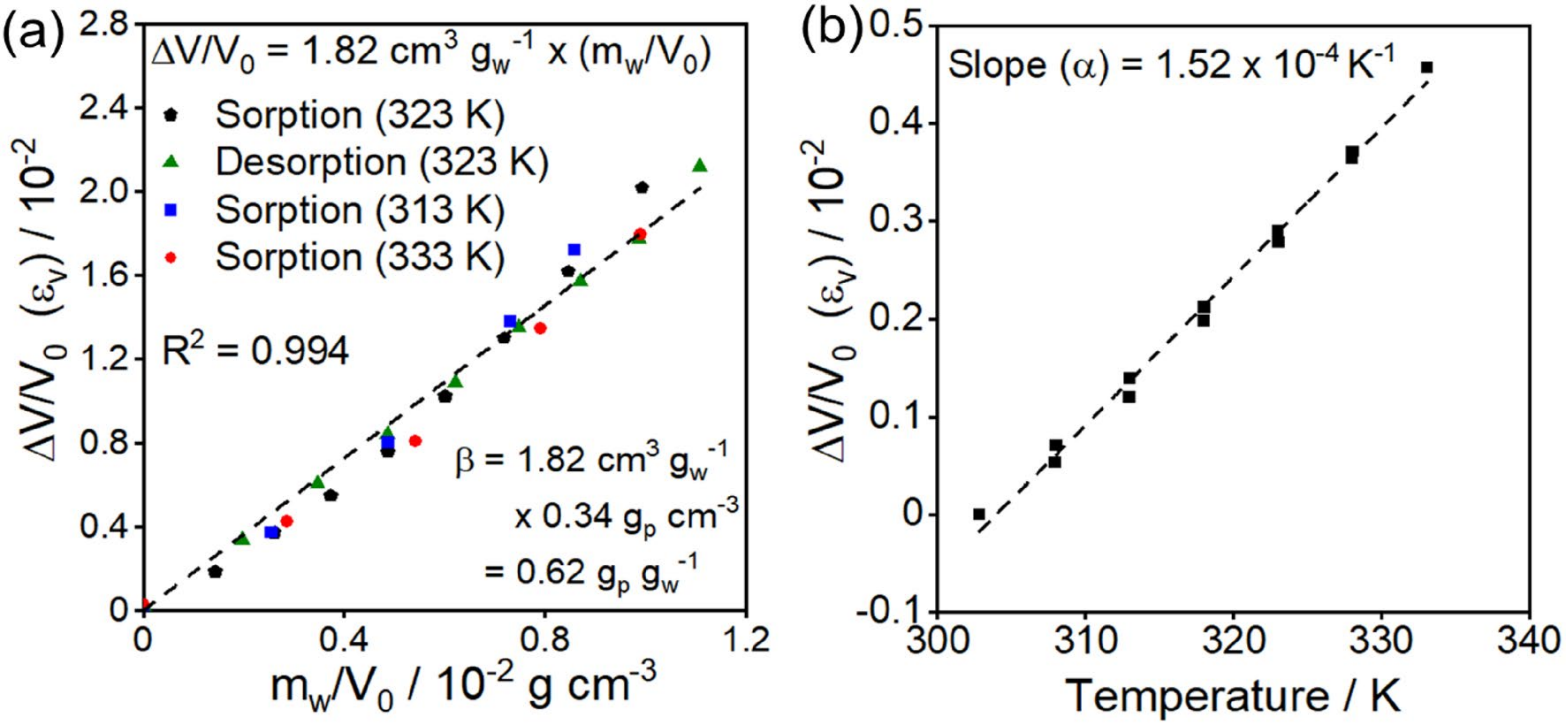
(**a**) Volumetric strain as a function of mass uptake per volume to find the hygroscopic expansion coefficient $\beta =0.62\, {{\text{g}}}_{{\text{p}}}/{{\text{g}}}_{{\text{w}}}$. (**b**) Volumetric strain as a function of temperature to find the thermal expansion coefficient, $\alpha =1.52\times {10}^{-4} \,{{\text{K}}}^{-1}$. See Sect. S2 of the Supporting Information for more details.

Equations (5)–(7) and (11) describe the sorption dynamics for diffusive transport in a material with multilayer adsorption at discrete sites in a polymer where site concentrations are kinetic functions of the volumetric strain. There are seven fitted parameters, ${L}_{0}, \gamma , D, b, \omega , k,$ and $n$, while the thermal and hygroscopic expansion coefficients $\alpha$ ($1.52\times {10}^{-4} {{\text{K}}}^{-1}$) and $\beta$ ($0.62\, \rm g_{\rm p}\,\rm g_{\rm w}^{-1}$) are measured from the data shown in Fig. 7. The seven fitted parameters are estimated by fitting analytical approximations of the dynamic model (see “Fitting dynamic model using analytical approximations” section for details) to the experimental data in Fig. 5 by the least-squares method. A temporal boundary condition is added to ensure the initial and final dry states have equal site concentrations, which deviate from the equilibrium concentration. The fit is given by the solid green line in Fig. 5a and by the solid lines in Fig. 5b. Parameter estimates are reported in Table 1.

**Table 1:** Fitted parameters to dynamic sorption data at 323 K.

Parameters	Fits
${b}^{\ddagger }(T=323 {\text{ K}})$	$3.36$
${\omega }^{\ddagger }(T=323 {\text{ K}})$	$0.435$
${L}_{\infty }\left(T=323 K, {c}_{{\text{eq}}}=0\right) \text{/} {\text{ mg}} {{\text{g}}}_{{\text{p}}}^{-1}$	$20.8$
$\gamma {/} {{\text{g}}}_{{\text{w}}} {{\text{g}}}_{{\text{p}}}^{-1}$	$0.411$
${k}^{\dag} \text{/} {s}^{-1}$	$6.02 \times {10}^{25}$
$n$	$12.9$
$D \text{/} {{\text{cm}}}^{2}\, {{\text{s}}}^{-1}$	$3.87\times {10}^{-7}$

^†^$k$ is given in units of inverse time assuming that ${L}_{\infty }$ and $L$ in Eq. (8) are rigorously normalized by the unit reference concentration 1 ${{\text{g}}}_{{\text{w}}}\, {{\text{g}}}_{{\text{p}}}^{-1}$.

^‡^Pure liquid water reference state (or reference pressure of ${P}^{{\text{o}}}={P}^{{\text{sat}}}$).

**Figure 8:**
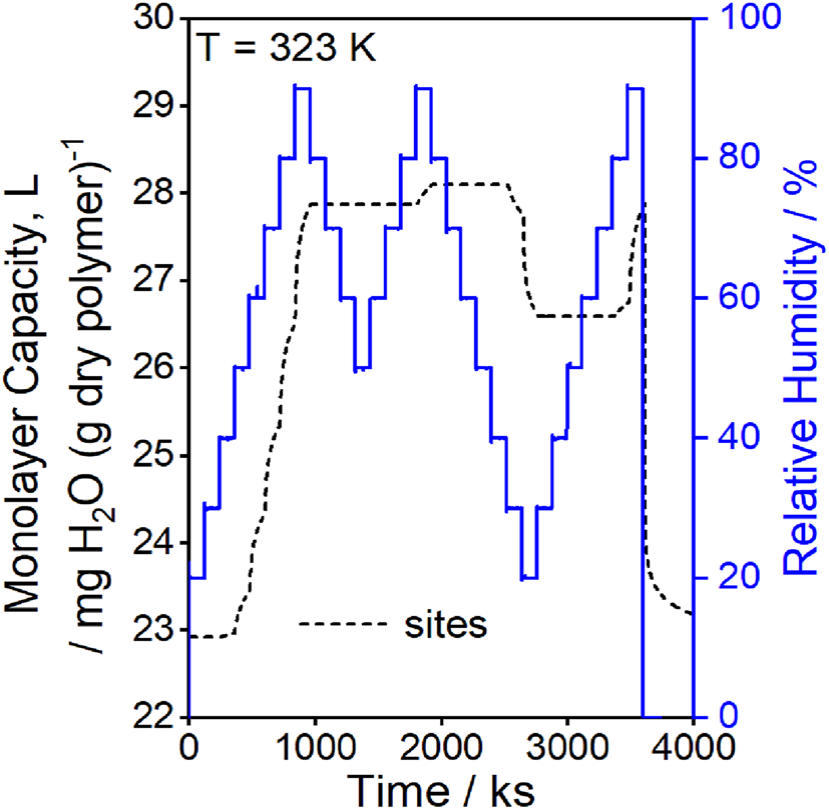
Model predicted monolayer capacity (site concentration) as a function of time for the multi-loop hysteresis data in Fig. 5.

The swelling GAB model is in good agreement with the experimental data in Fig. 5 and captures both the diffusion and relaxation regimes of sorption, as highlighted in the inset of Fig. 5a for the 60% RH sorption step. The model also captures the desorption for the 90 to 0% RH step at the end of the experiment which exhibits only diffusion-controlled outgassing. The hysteresis observed with varying minimum relative humidities are well-described by the model, as shown in Fig. 5b. Model predictions for the time-evolution of site concentration ($L$) by Eq. (11) are reported in Fig. 8. Because the slow kinetics prevent relaxation to an equilibrium state, the initial site concentration of ~ 23 mg g^−1^ determined by the temporal boundary condition is greater than the equilibrium concentration of 20.8 mg g^−1^. The 23 mg g^−1^ monolayer capacity corresponds to ~ 30% accessibility of urethane groups (see “Synthesis of polyurethane foam” section), which is within the range found for other polyurethanes in the literature^35^.

Confidence t-intervals are not given in Table 1 because temporal data are not independent, however inspection of the eigenvalues and eigenvectors of the Hessian matrix (see Sect. S3 of the Supporting Information) indicate that $k$ and $n$ are weakly estimated from the experimental data and are highly correlated, while other parameters are estimated with higher confidences, with ${L}_{0}$ as the parameter with the highest confidence. Equation (11) is required to fit the dynamic data, but several sufficiently large values of $n$ give similar fits, as shown in Fig. S5.

The kinetics and thermodynamics of water sorption in glassy polyurethane are facilely described by two simultaneously occurring phenomena: the diffusion of water to accessible hydrogen bonding sites and the kinetic change in the concentration of these available sites due to swelling. While full dynamic models describe both the dynamics and apparent sorption equilibria, it is useful to have simplified tractable models for describing sorption isotherms at multiple temperatures. Recent models describing substantially similar hysteresis mechanisms on amorphous cellulose proposed fixing the concentration of accessible sites as constant during desorption^5^. Such procedures simplify parameter estimation techniques but do not capture the decreasing site concentrations during desorption. In contrast, the chemo-mechanical model presented in this work is fit directly by assuming diffusive quasi-equilibrium is reached such that $\overline{\theta }\left(x,t\right)\approx {\overline{\theta }}_{{\text{eq}}}$ and $L\left(x,t\right)\approx L(t)$, and only Eqs. (7) and (11) are needed to fit the isotherms.

**Figure 9:**
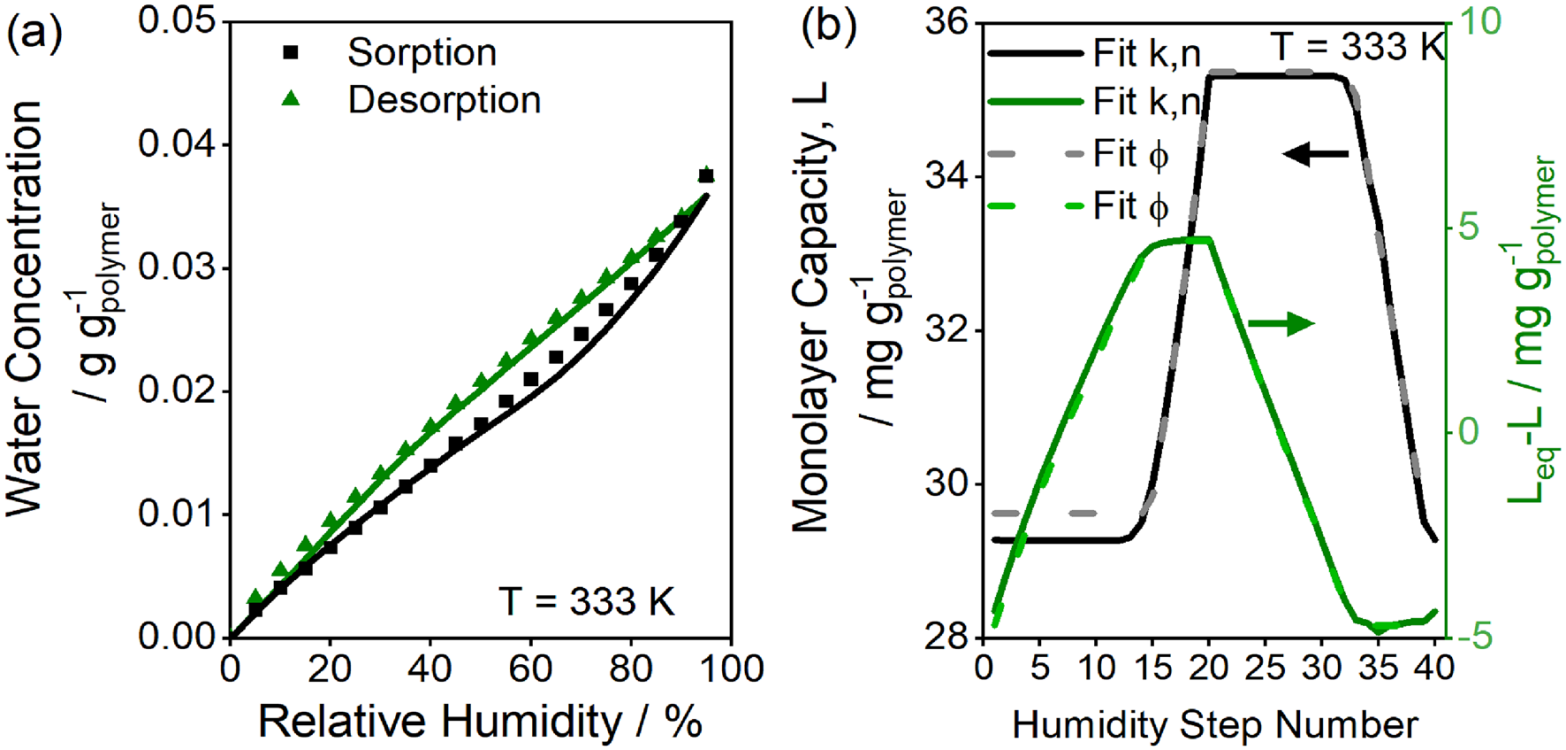
(**a**) Kinetic fit (solid lines) and max displacement fit (dashed lines) for water sorption and desorption on glassy polyurethane at 333 K using multi-temperature parameters reported in Table 2. Both fit methods overlay. (**b**) The site concentration (black/grey) and deviation from equilibrium (green) for kinetic fit (solid lines) and displacement fit (dashed lines) as a function of humidity step number, where 1–20 correspond to sorption steps and 21–41 correspond to desorption steps.

These equations were fit to experimentally measured sorption isotherms from 298 to 333 K with varying equilibration times, with parameter estimates reported in Table 2 and the 333 K isotherm fit reported in Fig. 9a. The temperature dependence of equilibrium constants is described by the van’t Hoff equation with enthalpy change of sorption as a fitted parameter, e.g., Eq. (12):

**Table 2:** Fitted parameters to 298–333 K sorption and desorption isotherms with 95% confidence t-intervals.

Parameters	Fit $k$ and $n$ (kinetic model)	Fit $\phi$ (max displacement model)
$b(T=298 {\text{ K}})$	$2.68 \pm 0.66$	$2.68\pm 0.65$
$\omega (T=298 {\text{ K}})$	$0.375 \pm 0.067$	$0.375\pm 0.067$
${L}_{\infty }\left(T=298 K, {c}_{{\text{eq}}}=0\right) \text{/} {{\text{g}}}_{{\text{w}}} \,{{\text{g}}}_{{\text{p}}}^{-1}$	$0.0213\pm 0.0030$	$0.0213\pm 0.0030$
$\gamma \text{/} {{\text{g}}}_{{\text{w}}} \,{{\text{g}}}_{{\text{p}}}^{-1}$	$0.68\pm 0.15$	$0.68\pm 0.14$
${k}^{\dag} \text{/} {s}^{-1}$	$1.6\times {10}^{23}\pm 4.8\times {10}^{24}$	–
$n$	$13.2\pm 5.9$	–
$\Delta {H}_{b}$^‡^$\text{/} \mathrm{kJ }\,{{\text{mol}}}^{-1}$	$-14.2\pm 1.9$	$-14.2\pm 1.9$
$\Delta {H}_{\omega }$^‡^$\text{/} \mathrm{kJ }\,{{\text{mol}}}^{-1}$	$-1.5\pm 1.2$	$-1.5\pm 1.2$
$\phi \text{/} {{\text{g}}}_{{\text{w}}}\, {{\text{g}}}_{{\text{p}}}^{-1}$	*–*	$0.0047\pm 0.0011$

^†^$k$ is given in units of inverse time assuming that ${L}_{\infty }$ and $L$ in Eq. (8) are rigorously normalized by the unit reference concentration 1 ${{\text{g}}}_{{\text{w}}} {{\text{g}}}_{{\text{p}}}^{-1}$.

^‡^Pure liquid water reference state (or reference pressure of ${P}^{{\text{o}}}={P}^{{\text{sat}}}$). Subtract the heat of condensation of water to convert to ideal gas reference state.

12 $$b\left(T\right)=b\left(T=298 {\text{ K}}\right){\text{exp}}\left(-\frac{\Delta {H}_{b}}{R}\times \left(\frac{1}{T}-\frac{1}{298 {\text{ K}}}\right)\right).$$

The sorption enthalpy for multi-molecular sorption ($\Delta {H}_{\omega }$) is closer to zero than monomolecular sorption ($\Delta {H}_{b}$), consistent with a more liquid-like ($\Delta H=0$) sorption state with increasing number of water molecules.

The confidence intervals on $k$ and $n$ are large, indicating again that a range of parameters suitably describe the site-change kinetics and that a reduced parameter model can capture the same experimental data. As illustrated in Fig. 9b, the kinetic term in Eq. (11) primarily ensures that the difference between ${L}_{\infty }$ and $L$ stays within a range $\pm \phi$, where $\phi \sim 5 \mathrm{\:mg\:}{{\text{g}}}^{-1}$. The site concentration does not increase or decrease until $\left|{L}_{\infty }-L\right|\sim \phi$. When $\left|{L}_{\infty }-L\right|<\phi$, no change in $L$ occurs, but if $\left|{L}_{\infty }-L\right|>\phi$ the kinetics then increase or decrease $L$ until the difference is equal to $\phi$. The prohibitively slow kinetics as $\left|{L}_{\infty }-L\right|$ tends towards zero causes this deviation from equilibrium to approach roughly a constant value of $\phi$ at laboratory timescales. Thus, for describing the equilibrium points, the kinetics are approximated by solving Eq. (10) for ${L}_{\infty }=L\pm \phi$ (Eq. 13):

13 $${L}_{n}=\left\{\begin{array}{ll}{L}_{n-1}& {\text{ if}}\; \left|{L}_{\infty }-{L}_{n-1}\right|<\phi \\ \frac{{L}_{0}(T)-\phi }{1-\gamma \beta {\overline{\theta }}_{{\text{eq}},n}}& \quad {\text{ if}} \;{L}_{\infty }-{L}_{n-1}>\phi \\ \frac{{L}_{0}\left(T\right)+\phi }{1-\gamma \beta {\overline{\theta }}_{{\text{eq}},n}}& \quad {\text{ if}} \; {L}_{\infty }-{L}_{n-1}<-\phi \end{array}\right.,$$

where ${L}_{n}$ is the site concentration at the end of humidity step *n*, ${L}_{n-1}$ is the site concentration at the end of the previous step, and ${\overline{\theta }}_{{\text{eq}},n}$ is ${\overline{\theta }}_{{\text{eq}}}$ during humidity step *n*. Equation (13) changes $L$ only if the driving force is greater than $\phi$. When ${L}_{\infty }-{L}_{n-1}>\phi$, then ${L}_{n}$ is *increased* so ${L}_{\infty }-{L}_{n}=\phi$, and if ${L}_{\infty }-{L}_{n-1}<-\phi$, ${L}_{n}$ is *decreased* such that ${L}_{\infty }-{L}_{n}=-\phi$. Equation (13) provides a simple algebraic approach for fitting the swelling GAB model to sorption–desorption isotherms. We refer to this approach as the “Max Displacement Model” because bounds are imposed on $L$ to ensure it stays within ${L}_{\infty }\pm \phi$.

**Figure 10:**
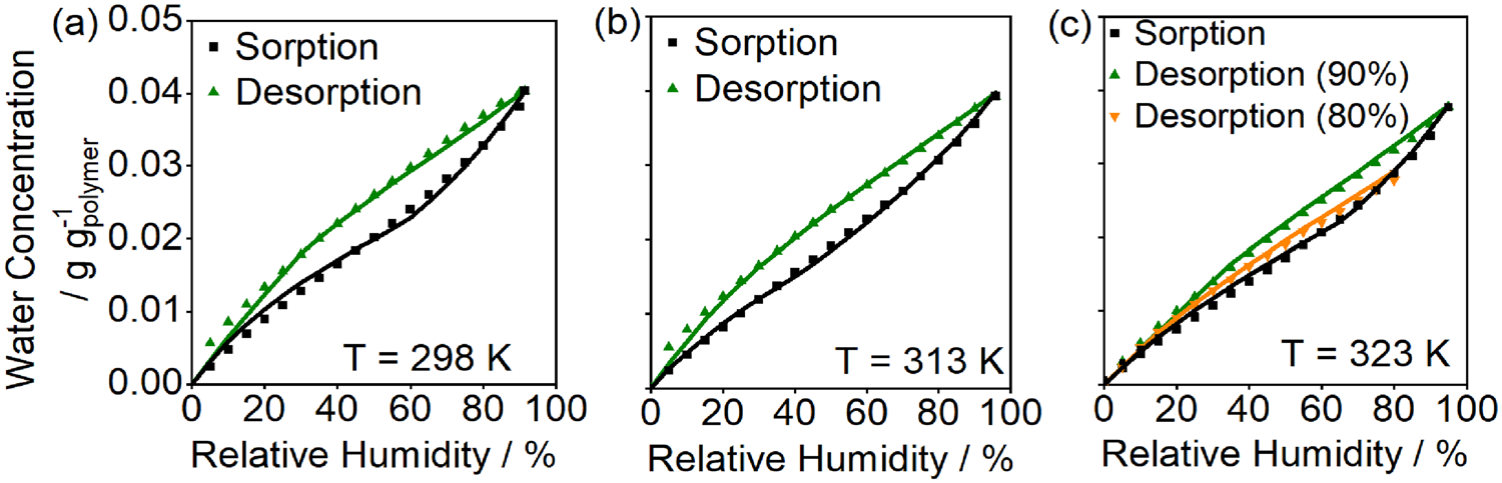
Displacement model fits for (**a**) 298 K, (**b**) 313 K, and (**c**) 323 K for model parameters presented in Table 2. The displacement model captures sorption and desorption hysteresis at all temperatures and the desorption curve at either 90% or 80% RH at 323 K.

**Figure 11:**
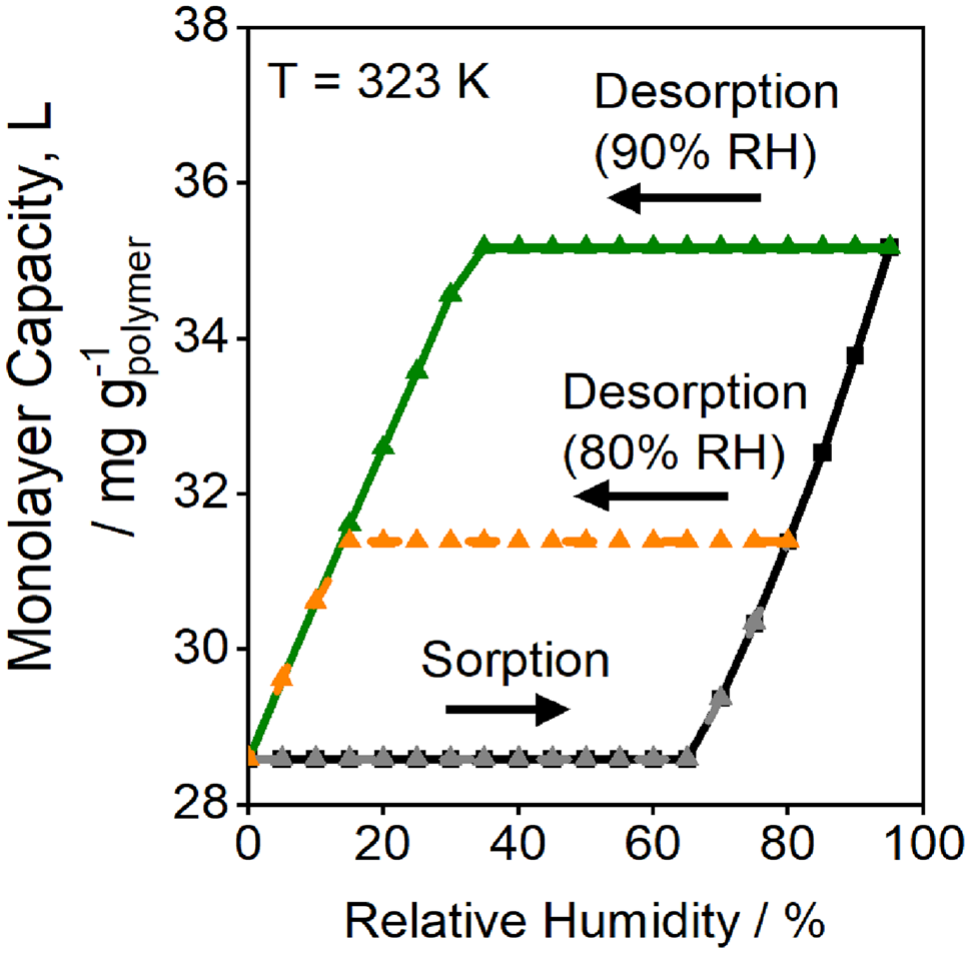
Monolayer capacity of water (site concentration, *L*) as a function of relative humidity during sorption and desorption at 323 K for the max displacement model fit. The site concentration during desorption at 80% RH is lower than that at 90% RH, causing the hysteresis loop to be smaller as shown in Fig. 10c.

The equilibrium isotherms were refit with Eq. (13) replacing the kinetics described in Eq. (11). The initial dry site concentration is taken as ${L}_{1}={L}_{0}\left(T\right)+\phi$ found by substitution of ${\overline{\theta }}_{{\text{eq}},n}=0$ into Eq. (13). This fit gives comparable parameters to the kinetic fit, as shown in Table 2, with the exception that the parameter $\phi$ has a reasonable 95% confidence t-interval of ~ 25%, indicating the parameter was estimable from the model fit. Comparison of model fits for water concentration and accessible site concentration in Fig. 9 demonstrate that Eq. (13) accurately approximates the kinetic equations for isotherm values. The fits of the other isotherms are shown in Fig. 10. The model captures hysteresis from 298 to 333 K and describes varying hysteresis loops, as illustrated in Fig. 10c. Site concentrations differ during sorption and desorption and form a closed loop, as shown in Fig. 11 for the 323 K isotherm. The model captures hysteresis loops of varying maximum humidities because the monolayer capacity, $L$, depends on the entire sorption history of the polymer (Fig. 11).

## Conclusion

Water sorption on glassy polyurethane exhibits hysteresis because of the slow relaxation of the polymer chains to equilibrium. The model developed in this work couples the relaxation dynamics to the resulting hysteresis. These relaxation dynamics are functions of polymer state variables, allowing the model to describe the non-Fickian dynamics at all conditions, rather than each humidity step individually. A simplified model form that estimates strain-dependent site concentrations using algebraic equations is easily employed to simultaneously fit multiple isotherms with varying sized hysteresis loops while still capturing the effect of complex sorption histories. These tractable models are easily extended to describe sorption hysteresis in other glassy polymers, especially those with groups amenable to hydrogen bonding with water.

## Methods

### Moisture uptake experiments

The dynamic mass uptake and outgassing of water on glassy polyurethane foam (15 × 20 × 0.48–6.0 mm) were measured for 0–95% relative humidity (± 0.01% RH regulation accuracy, ± 1% RH calibration accuracy) at 298–353 K (± 0.01 K) with a temporal resolution of 1–60 s^45^. Samples were prepared following the same synthesis procedure but the 0.48 mm thick sample was from a different lot than all other samples and exhibited slightly higher water sorption. Typically data were collected on an IGAsorp (Hiden Isochema) equipped with a microbalance (± 0.05 $\upmu$g) using a 2.0 mm thick sample^45^. Humid nitrogen is delivered to the sample at a flow rate of 4.167 cm^3^ s^−1^ by mixing dry and saturated nitrogen streams. The dynamic uptake of samples of varying thicknesses was assessed on a multi-sample dynamic vapor sorption instrument (Mettler Toledo, SPSx-1µ High Load) equipped with a microbalance (± 5 $\upmu$g) and temperature (± 0.1 K) and humidity (± 0.6% RH) controls with a temporal resolution of ~ 600 s^46^. Humid nitrogen is generated by mixing dry and saturated nitrogen streams and recirculated internally using fans to ensure good mixing. Liquid nitrogen is used as a gaseous nitrogen source and deionized water is used for both instruments.

### Thermomechanical and chemo-mechanical analysis

Thermal and hygroscopic expansion were assessed using a thermomechanical analyzer (TMA 402 F1 Hyperion®, Netzsch) equipped with a modular humidity generator (MHG32, ProUmid) and a copper furnace^47,48^. The linear strain (${\varepsilon }_{{\text{L}}}$) was measured by assessing the length (1.25 nm resolution) of a cylindrical polyurethane sample (15.0 mm length × 5.0 mm diameter) by a pushrod exerting a linear force of 0.200 N under a flowing N_2_ (Airgas, $\ge 99.999\%$ purity, 0.33 cm^3^ s^−1^) environment with a specified humidity (0–85%, ± 0.8%) from 303 to 333 K (± 0.1 K)^47,48^. The volumetric strain (${\varepsilon }_{{\text{V}}}$) was calculated from the measured linear strain under the assumption that the linear strain is small, the sample is isotropic, and the force exerted by the pushrod is negligible, such that ${\varepsilon }_{{\text{V}}}=3{\varepsilon }_{{\text{L}}}$. The stress–strain relationship was measured at 313 K at 0% and 60% relative humidity by ramping the linear compressive stress exerted by the pushrod at a rate of 0.3 Pa s^−1^.

### Synthesis of polyurethane foam

The glassy polyurethane foam is Series BKC 44306 produced by the National Nuclear Security Administration’s Kansas City Plant, operated by Honeywell Federal Manufacturing & Technologies, LLC^49^. It is formulated from a 40.3 to 59.7 weight ratio of the R- and T-components summarized in Table 3. The polyurethane is foamed at the Kansas City Plant by mixing R- and T-components with an impeller-type electric mixer at 1500–1800 rpm and pouring 200 g of the mixture into a pre-heated mold (52 °C), taking care to avoid air entrapment. A combination of a shot weight and a closed mold are used to limit the foam expansion during curing. The foam is allowed to gel at room temperature for 20–30 min before curing in an oven at 163 °C for 4 h. The mold is removed from the oven and allowed to cool to room temperature before removing the cured polymer.

**Table 3:** Composition by weight of polyurethane components^49^.

Material	Parts by weight
R-component	40.3
Voranol 490 (polyol)	100
Water	0.8
Dabco DC-197 (surfactant)	1.0
TMPDA (catalyst)	0.7
T-component	59.7
PAPI 27 (isocyanate)	100

Samples of 2-, 4-, and 6-mm thicknesses are taken from the foam bulk while the 0.48 mm thickness sample is taken from the outer edge of the material (see Table 4 for precise dimensions and masses). The isocyanate groups are 31.4–31.8 wt% NCO groups, which corresponds to 18.9 wt% NCO groups in the final pre-polymerization mixture. We approximate the final urethane group concentration by assuming that NCO groups react with water to produce CO_2_ and the remaining NCO groups react to form urethane groups with 1:1 stoichiometry. This corresponds to 4.49 mmol g^−1^ urethane groups in the final polymer or a maximum water monolayer capacity of 77.7 mg g^−1^. The resulting polyurethane has a glass transition temperature of $\sim 413 {\text{ K}}$ (see Sect. S1 of the Supporting Information).

**Table 4:** Sample dimensions and masses used for water sorption experiments.

Thickness/mm	Height/mm	Width/mm	Mass/mg
0.48	20.00	15.00	0.063
2.05	19.80	15.01	0.214
2.04	20.06	15.02	0.194
4.06	20.00	15.06	0.417
6.05	19.96	14.83	0.635

### Estimating diffusivity for each humidity step in Fig. 6

The diffusivity was estimated by fitting the analytical function for mass uptake with both an exponential decay boundary condition and a Berens–Hopfenberg (B–H) kinetic term, modified from Burgess et al.^43^:

14 $${M}_{\rm tot}\left(t\right)={M}_{\rm F}\left[1-{\rm exp}\left(-\frac{t}{{\tau }_{\rm S}}\right){\left(\frac{4{\tau }_{\rm S}D}{{h}^{2}}\right)}^{1\text{/}2}{\rm tan}\sqrt{\frac{{h}^{2}}{4{\tau }_{\rm S}D}}-\frac{8}{{\pi }^{2}}\sum \limits_{n=0}^{\infty }\frac{{\rm exp}\left(-D{\left(2n+1\right)}^{2}{\pi }^{2}\frac{t}{{h}^{2}}\right)}{{\left(2n+1\right)}^{2}\left(1-{\left(2n+1\right)}^{2}\left(\frac{{\tau }_{\rm S}D{\pi }^{2}}{{h}^{2}}\right)\right)}\right]+{M}_{\rm{B-}\rm H}\left(1-{\rm exp}\left(-\frac{t}{{\tau }_\text{B-H}}\right)\right),$$

where ${\tau }_{{\text{S}}}$ is the timescale for the boundary condition to reach equilibrium, $D$ is the diffusivity, $h$ is the sample thickness, ${\tau }_{\text{B-H}}$ is the timescale for the B–H kinetic term, and ${M}_{{\text{F}}}$ and ${M}_{\text{B-H}}$ are the relative mass contribution of Fickian diffusion and B–H kinetics, respectively. This equation was fit to the dynamic uptake for each humidity step separately using the method of least squares to estimate the diffusivities reported in Fig. 6.

### Fitting dynamic model using analytical approximations

Initially, the rate of water uptake is diffusion controlled (see Figs. 2, 3) and $L\left(x,t\right)\approx {L}_{i}$, where ${L}_{i}$ is the initial concentration of sites available at the start of an RH step. Thus, at early times $\partial L\text{/}\partial t\approx \partial L\text{/}\partial x\approx 0$, and the transport equation (Eq. (5)) simplifies to Eq. (15).

15 $${L}_{i}\frac{\partial \overline{\theta }}{\partial t}=D{L}_{i}\frac{{\partial }^{2}\overline{\theta }}{\partial {x}^{2}}.$$

At longer times, $\overline{\theta }\left(x,t\right)\approx {\overline{\theta }}_{{\text{eq}}}$ because the sample is in diffusive quasi-equilibrium, but the water concentration continues to increase as $L$ increases (Eq. 11). By summing the diffusive and site-concentration kinetic contributions, we derive an expression for describing the uptake dynamics as Eq. (16):

16 $$\frac{{\partial c}}{{\partial t}} \approx \mathop {L_{i} \frac{{\partial \bar{\theta }\left( {x,t} \right)}}{{\partial t}}}\limits_\text{Diffusion control} + \mathop {\bar{\theta }_{{{\text{eq}}}} \frac{{\partial L\left( t \right)}}{{\partial t}}}\limits_{{\text{Site-concentration control}}} ,$$

where $\overline{\theta }$ is a function of $x$ and $t$ while $L$ is approximated as a function of only $t$ because uptake is initially diffusion-controlled. This approximation is validated at the end of this derivation in “Finite difference methods for solving partial differential equations” section. The change in mass water per mass polymer for a single step, $M\left(t\right)$, is found by integrating Eq. (10) over the time of the humidity step over the thickness of the sample (Eq. 17):

17 $$M\left(t\right)=\frac{1}{h}{\int }_{0}^{h}{\int }_{0}^{t}\frac{\partial c}{\partial t} {\text{ d}}t {\text{ d}}x\approx \frac{1}{h}{\int }_{0}^{h}\left({\int }_{0}^{t}{L}_{i}\frac{\partial \overline{\theta }}{\partial t}{\text{d}}t+{\int }_{0}^{t}{\overline{\theta }}_{{\text{eq}}}\frac{\partial L}{\partial t}{\text{d}}t\right){\text{d}}x ={L}_{i} \frac{1}{h}{\int }_{0}^{h}\left(\overline{\theta }\left(x,t\right)-\overline{\theta }\left(x,t=0\right)\right) {\text{ d}}x+{\overline{\theta }}_{{\text{eq}}}\left(L\left(t\right)-{L}_{i}\right)={M}_{{\text{D}}}\left(t\right)+{M}_{{\text{k}}}\left(t\right),$$

which is then separated into the contribution due to diffusion, ${M}_{{\text{D}}}\left(t\right)$, and the contribution due to site-concentration change kinetics, ${M}_{{\text{k}}}\left(t\right)$. The solution for the total mass change in the diffusion-controlled regime is given by solving Eq. (9) and averaging the local sorbed concentration ${L}_{i}\overline{\theta }(x,t)$ over the thickness of the sample (from Crank^50^):

18 $${M}_{{\text{D}}}\left(t\right)=\left({\overline{\theta }}_{{\text{eq}}}-{\overline{\theta }}_{i}\right){L}_{i}\left(1-\sum \limits_{m=0}^{\infty }\frac{8}{{\left(2m+1\right)}^{2}{\pi }^{2}}{\text{exp}}\left(-\frac{{\left(2m+1\right)}^{2}{\pi }^{2}D}{{h}^{2}}t\right)\right),$$

assuming constant concentration $$\left(\overline{\theta }\left(x=0,t\right)=\overline{\theta }\left(x=h,t\right)={\overline{\theta 
}}_{{\text{eq}}}\right)$$ and no-flux ($$\partial \overline{\theta }\text{/}\partial x(x=h\text{/}2,t))$$ boundary conditions and a uniform concentration $$\left(\overline{\theta }\left(x,t=0\right)={\overline{\theta }}_{i}\right)$$ initial condition. The contribution to total mass change by increasing site concentration is found by solving Eq. (11) with a $$L\left(t=0\right)={L}_{i}$$ initial condition, which is given for $$n\ne 1$$ as Eqs. (19)–(22):

19 $$L\left(t\right)=\left\{\begin{array}{c}\begin{array}{cc}-\frac{A}{B}+\frac{(B\left(1-n\right){\left(p+kt\right))}^{1\text{/}\left(1-n\right)}}{B}& \forall\; {L}_{\infty }>{L}_{i}\end{array}\\ \begin{array}{cc}-\frac{A}{B}-\frac{(B\left(1-n\right){\left(p+kt\right))}^{1\text{/}\left(1-n\right)}}{B}& \forall \;{L}_{\infty }<{L}_{i}\end{array}\end{array}\right.,$$

20 $${M}_{k}\left(t\right)={\overline{\theta }}_{{\text{eq}}}\left(L\left(t\right)-{L}_{i}\right),$$

21 $$p=\frac{{\left|B\left({L}_{i}+\frac{A}{B}\right)\right|}^{1-n}}{B(1-n)},$$

22 $$\begin{gathered} A \equiv L_{0} + \gamma \alpha \left( {T - T_{{{\text{ref}}}} } \right) \hfill \\ B \equiv \left( {\gamma \beta \overline{\theta }_{{{\text{eq}}}} - 1} \right), \hfill \\ \end{gathered}$$

where $p$ is the constant of integration and $A$ and $B$ are variables defined in Eq. (22). Note that ${\overline{\theta }}_{{\text{eq}}}$, ${L}_{\infty }$, ${\overline{\theta }}_{i}$, and ${L}_{i}$ correspond to the equilibrium and initial value for each humidity step and that $t=0$ for the start of each step. In this analytical approximation, $L\left(t\right)$ is invariant with $x$ under the assumption that changes in $L$ are driven by kinetics on timescales that are much larger than those for diffusion. At infinite time scales, the site concentration converges to ${L}_{\infty }$ given by Eq. (23) when $1>\gamma \beta {\overline{\theta }}_{{\text{eq}}}$ and diverges to infinity otherwise. We verify the accuracy of these analytical approximations in “Finite difference methods for solving partial differential equations” section by comparing a numerical integration of Eqs. (5)–(11) without any approximations on the fast and slow timescales

23 $${L}_{\infty }=\frac{\left({L}_{0}+\gamma \alpha \left(T-{T}_{{\text{ref}}}\right)\right)}{1-\gamma \beta {\overline{\theta }}_{{\text{eq}}}}.$$

### Finite difference methods for solving partial differential equations

The Crank–Nicolson finite difference method^51^ was used to solve a system of coupled partial differential equations to estimate the water sorption dynamics in glassy polyurethane. The spatial mesh consisted of 101 equidistance points and time steps of ${10}^{-4} {h}^{2}/4D$ were used, where h is the sample thickness and $D$ is the diffusivity of water in the sample.

**Figure 12:**
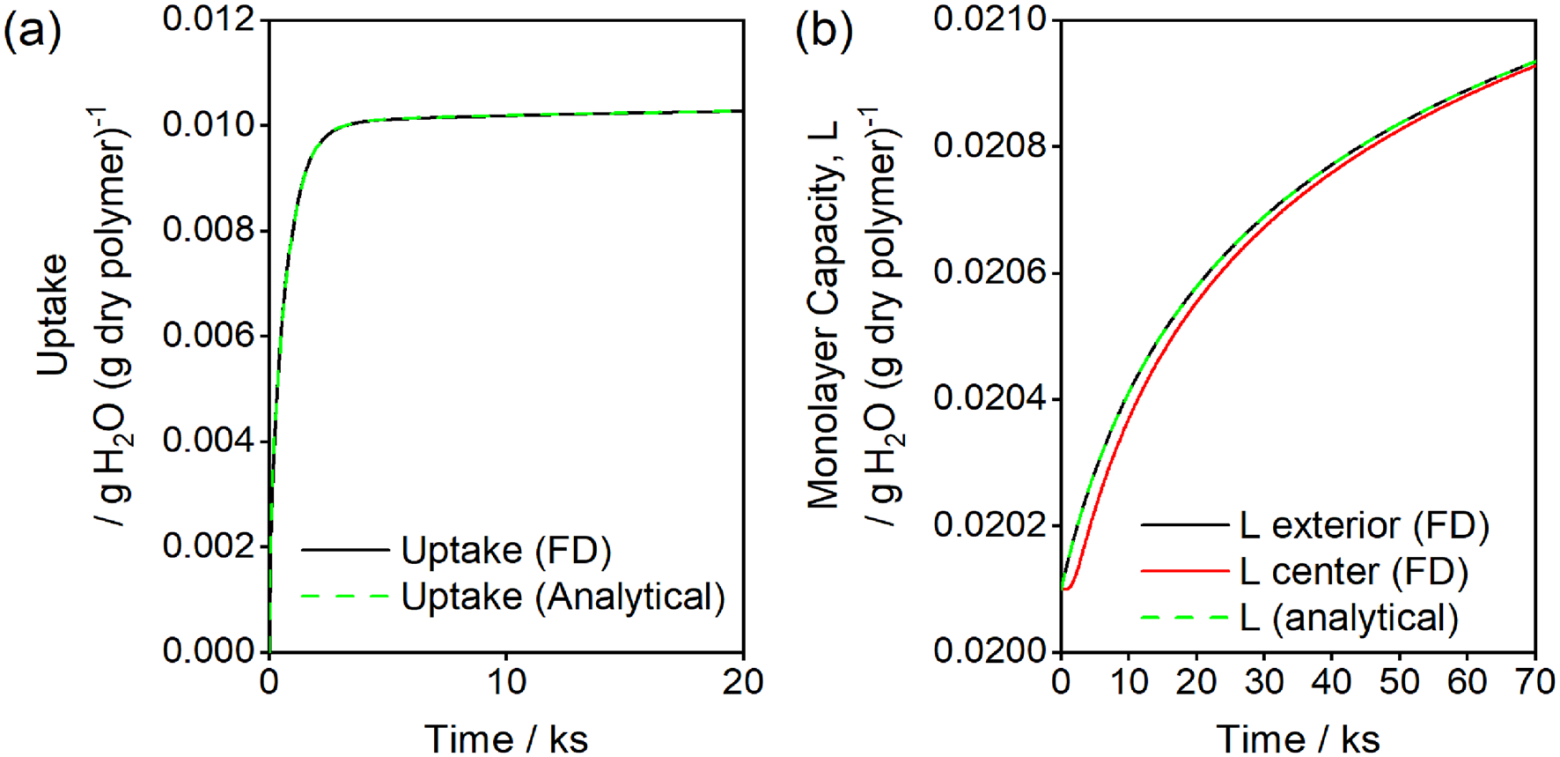
(**a**) Comparison of uptake from finite difference (FD) calculations (black line) of full partial differential equations (Eqs. 1–8) to the analytical approximations (dashed green line, Eqs. 15–23) for an RH step from 0 to 20%. (**b**) Comparison of monolayer capacity at the exterior and center of the 0.48 mm polyurethane slab at the exterior (black line) and center (red line) as calculated by finite difference methods. The analytical approximation assumes $L$ is uniform throughout the material and equal to L exterior from the finite difference method. For this simulation, $D=2.3 \times {10}^{-7} {{\text{ cm}}}^{2}{ {\text{ s}}}^{-1}, k=6.15\times {10}^{8}\, {{\text{s}}}^{-1}$, $b=3.77$, $\omega =0.46$, ${L}_{i}=0.0185 {g}_{{\text{w}}}\, {g}_{{\text{p}}}^{-1}$, $\gamma =0.36$, $n=6.08$.

The analytical model used to estimate parameters and fit the experimental data approximates the coupled partial differential equations given in Eqs. (1)–(7). To verify that this analytical model is an accurate approximation of Eqs. (1)–(7), the partial differential equations are numerically solved by finite difference methods and compared to the analytical approximations for a 0 to 20% RH step in Fig. 12. There is a negligible difference between the models for describing the uptake dynamics of water, as shown in Fig. 12a. The analytical approximation assumes a uniform monolayer capacity, $L$, which is a sufficiently close approximation of the analytical solution, as shown in Fig. 12b. In the numerical solution, there is a delay between increasing site concentrations at the external surface and that for the slab center, but this lag is inconsequential for the thin slabs used in this experiment.

### Supplementary Information

Supplementary Information 1.

Supplementary Information 2.

## Data Availability

All data generated or analyzed during this study are included in this published article (and its Supplementary Information files).

## List of symbols

$a_{{\text{w}}} \left[ - \right]$: Thermodynamic activity of water. In this work, a pure liquid water reference is taken, such that the activity is equal to the relative humidity ($P_{{\text{w}}} /P_{{\text{w}}}^{{{\text{sat}}}}$)
$b \left[ - \right]$: Equilibrium constant for monolayer water sorption at a urethane site
$c \left[ {{\text{g H}}_{2} {\text{O/g polymer}}} \right]$: Sorbed water mass concentration per mass polymer (function of $x$ and $t$)
$D \left[ {{\text{cm}}^{2} {\text{ /s}}} \right]$: Mass diffusivity of water in polyurethane
$k \left[ {{\text{s}}^{ - 1} } \right]$: Rate constant for rate of change of $L$
$$h \left[ {{\text{cm}}} \right]$$: Sample thickness
${\Delta }H_{b} \left[ {\text{ kJ/mol}} \right]$: Sorption enthalpy for forming water monolayer on urethane groups from pure liquid water reference state
${\Delta }H_{\omega } \left[ {\text{ kJ/mol}} \right]$: Sorption enthalpy for forming water multilayers on urethane groups from pure liquid water reference state
$L \left[ {{\text{g H}}_{2} {\text{O/g polymer}}} \right]$: Urethane group site concentration, expressed as a monolayer capacity for water. (function of $x$ and $t$)
$L_{i} { }\left[ {{\text{g H}}_{2} {\text{O/g polymer}}} \right]$: Urethane group site concentration at the start of a particular humidity step
$L_{n} \left[ {{\text{g H}}_{2} {\text{O/g polymer}}} \right]$: Urethane group site concentration in the previous humidity step for the max displacement model
$L_{\infty } \left[ {{\text{g H}}_{2} {\text{O/g polymer}}} \right]$: Urethane group site concentration after infinite relaxation time at a fixed volumetric strain (function of $x$ and $t$)
$M \left[ {{\text{g H}}_{{2}} {\text{O/g polymer}}} \right]$: Mass change that occurs during a single humidity step (function of time)
$M_{\rm{B-} \rm H} \left[ - \right]$: Relative mass uptake at equilibrium from the Beren-Hopfenberg kinetic term in Eq. (8) (*not* a function of time)
$M_{\rm D} \left[ \rm g\,H_{2} O\text{/} \rm g polymer\right]$: Mass change that occurs during the diffusion-controlled regime of a single humidity step (function of time)
$M_{{\text{F}}} \left[ - \right]$: Relative mass uptake at equilibrium from Fickian-diffusion control in Eq. (8) (*not* a function of time)
$M_{{\text{k}}} \left[ {{\text{g H}}_{{2}} {\text{O/g polymer}}} \right]$: Mass change that occurs when site-change-kinetics are controlling uptake rate (function of time)
$M_{{{\text{tot}}}} \left[ - \right]$: Relative total mass change as a sum of Fickian diffusion and Berens-Hopfenberg kinetics in Eq. (8) (function of time)
$n \left[ - \right]$: The order parameter for describing rate of change of $L$
$$R \left[ {\text{ J/mol/K}} \right]$$: Gas constant
$$t \left[ {\text{ s}} \right]$$: Time
$T [{\text{K]}}$ ($T_{{{\text{ref}}}} \left[ K \right]$): Absolute temperature (Absolute reference temperature)
$\omega \left[ - \right]$: Equilibrium constant for multilayer water sorption at a urethane site
$x \left[ {{\text{cm}}} \right]$: Spatial variable for depth in sample

## Greek symbols

$\alpha \left[ {{\text{K}}^{{-1}} } \right]$: Thermal expansion coefficient
$\beta \left[ {{\text{g polymer/g H}}_{2} {\text{O}}} \right]$: Hygroscopic expansion coefficient
$$\varepsilon_{{\text{v}}} \left[-\right]$$: Volumetric strain
$\gamma \left[ {{\text{g H}}_{2} {\text{O/g polymer}}} \right]$: First-order parameter describing how $L_{\infty }$ changes as a function of volumetric strain
$\mu \left[ {\text{ J/mol}} \right]$: Chemical potential (function of $x$ and $t$)
$\mu^{{\text{o}}} \left[ {\text{ J/mol}} \right]$: Reference state chemical potential
$\overline{\theta } \left[-\right]$: Average number of water molecules sorbed per accessible urethane group (function of $x$ and $t$)
$\overline{\theta }^{{\text{o}}} \left[-\right]$: Reference state for sorbed water moleculaes. Taken as 1.
$\overline{\theta }_{{{\text{eq}}}} \left[-\right]$: The average number of water molecules sorbed per accessible sorption site at equilibrium with the gas phase relative humidity.
$\phi \left[ {{\text{g H}}_{2} {\text{O/g polymer}}} \right]$: Displacement factor for the “Max Displacement Model” that sets the difference between $L$ and $L_{\infty }$ at laboratory timescales
