# Supplementary material for: A chemo-mechanical model for describing sorption hysteresis in a glassy polyurethane
Source: Sci Rep. 2024 Mar 7;14:5640. doi: 10.1038/s41598-024-56069-3 (PMC10920897; doi:10.1038/s41598-024-56069-3)
Supplement: Supplementary file 1 — Supplementary Information 1. [file 41598_2024_56069_MOESM1_ESM.docx]

**Supporting Information for “A chemo-mechanical model for describing sorption hysteresis in a glassy polyurethane”**

Brandon L. Foley,^1^ Sarah M. Matt,^1^ Stephen T. Castonguay,^1^ Yunwei Sun,^1^ Pratanu Roy,^1^ Elizabeth A. Glascoe,^1,*^ and Hom N. Sharma^1,2,Ψ^

^1^*Lawrence Livermore National Laboratory, 7000 East Avenue, Livermore, California 94550*

^2^*Currently at the U.S. Centers for Disease Control and Prevention (CDC), National Institute for Occupational Safety and Health (NIOSH), National Personal Protective Technology Laboratory (NPPTL).*

*Corresponding Author: E-mail: [glascoe2@llnl.gov](mailto:glascoe2@llnl.gov)

# S1. Discussion on Vrentas and Vrentas, non-equilibrium lattice fluid, and poromechanics hysteresis models

## Vrentas and Vrentas Model

There exist many models for capturing hysteresis over a wide range of chemical activities, each relying on different underlying phenomena. For example, the Vrentas and Vrentas^3^ model and the non-equilibrium lattice fluid (NELF)^4,5^ model assume that the polymer's specific volume differs during sorption and desorption. The chemical potential of species in the sorbed state are then functions of the polymer specific volume, leading to different sorbed concentrations during sorption and desorption. The model proposed by Vrentas and Vrentas assumes a negative excess volume of mixing in its derivation,^6^ which is in contrast to the positive excess volume of mixing observed for water sorption in the glassy polyurethane (Figure S2). Thus, we conclude that the Vrentas and Vrentas model is inappropriate for water sorption in glassy polyurethane.

## Non-equilibrium fluid lattice model

Non-equilibrium fluid lattice models treat the polymer density ($\rho_{p}$) as a state variable, like pressure and temperature, on which the sorbed chemical potential ($\mu_{w}^{\mathrm{NE}})$ depends, such that equality of chemical potential in the two phases is given by (eq. (S1)):

| $\mu_{w}^{\mathrm{NE}}\left( T,m_{w}\text{/}m_{p},\rho_{p} \right)=\mu_{w}^{\mathrm{gas}}\left( T,P_{w} \right)$ | (S1) |
| --- | --- |

where $m_{w}\text{/}m_{p}$ is mass water sorbed per mass polymer, $P_{w}$ is the partial pressure of water in the gas phase, and $\mu_{w}^{\mathrm{gas}}$ is the chemical potential of water in the gas phase. Based on the volumetric data reported in Figure S2b, it is reasonable to assume that when comparing sorption and desorption behavior at identical mass uptake, i.e., where $\left( m_{w}\text{/}m_{p} \right)_{\mathrm{ads}}=\left( m_{w}\text{/}m_{p} \right)_{\mathrm{des}}$, the polymer densities at these conditions are identical such that $\rho_{p,ads}=\rho_{p,des}$. To compare at identical mass uptakes, the partial pressure of water in the gas phase is necessarily different for sorption and desorption at identical mass uptakes, such that $P_{w,ads}\neq P_{w,des}$. It follows from eq. (S1) that during sorption and desorption, the equilibrium between gas phase and sorbed water is described by eqs. (S2) and (S3).

| $\mu_{w}^{\mathrm{NE}}\left( T,\left( m_{w}\text{/}m_{p} \right)_{\mathrm{ads}},\rho_{p,ads} \right)=\mu_{w}^{\mathrm{gas}}\left( T,P_{w,ads} \right)$ | (S2) |
| --- | --- |
| $\mu_{w}^{\mathrm{NE}}\left( T,\left( m_{w}\text{/}m_{p} \right)_{\mathrm{des}},\rho_{p,des} \right)=\mu_{w}^{\mathrm{gas}}\left( T,P_{w,des} \right)$ | (S3) |

Based on the discussion above, at identical mass uptakes, the chemical potential of water in the sorbed phase during desorption and sorption should be equal because the independent variables on the left-hand sides of eqs. (S2) and (S3) are all equal. However, at identical mass uptakes, the chemical potentials of water in the gas phase are different during sorption and desorption, since $P_{w,ads}\neq P_{w,des}$, and thus the right-hand sides of eqs. (S2) and (S3) are not equal. This contradiction suggests that the current non-equilibrium fluid lattice models are not appropriate for describing sorption hysteresis in glassy polyurethane. In glassy polyurethane, measurement of the volumetric strain simply represents an alternative means of assessing the moisture uptake by nature of the one-to-one correspondence between the two quantities. In order for non-equilibrium lattice fluid models to capture the hysteresis in glassy polyurethane, a state variable describing the accessibility of polyurethane groups would need to be added with a differential equation describing the non-equilibrium time-evolution of this variable, as is done in the swelling GAB model.

## Poromechanics Model

The third model we discuss here is a poromechanics model derived by Chen et al.^7^ for hysteresis during water sorption on amorphous cellulose based on insights from MD simulations, which we believe has a similar mechanism to hysteresis in glassy polyurethane.^2^ The reader is directed to the original work^7^ for the full derivation. In this section, we compare the swelling GAB model with the poromechanics model to highlight the key differences and similarities.

The poromechanics model assumes that a critical distance between cellulose chains, $R_{C}$, is required at hydrogen bonding sites for sorption to occur, and that there is a population distribution of these distances $R$. All sites with $R<R_{C}$ are too small for water to adsorb, and thus are defined as “closed,” while sites with $R>R_{C}$ are large enough for water to adsorb and are considered “open.” As the material swells due to moisture sorption, the distances $R$ increase such that a greater fraction of sites have $R>R_{C}$ and become open and accessible for water sorption. During desorption, it is assumed that all available sites remain open until the material is dried. The poromechanics model assumes that multiple water molecules can sorb at each Langmuir-like hydrogen-bonding site with an equilibrium described by eq. (S4):

| $n=\left\{ \begin{matrix} \frac{Ba_{w}}{1+Ba_{w}}n_{0}\left( 1+C\varepsilon_{v} \right) & \text{open sites} \\ 0 & \text{closed sites} \end{matrix} \right.$ | (S4) |
| --- | --- |

where $B$ (Pa^-1^) is the equilibrium constant for sorption,$a_{w}$is the activity of water, $n$ is the water concentration, $n_{0}$ is the sorption capacity at zero volumetric strain, and $(1+C\varepsilon_{v})$ is a factor that describes how the sorption capacity at each site increases as a function of the volumetric strain ($\varepsilon_{v}$) due to increasing porosity, where $C$ is a constant. The swelling GAB model describes eq. (S4) as:

| $n=\left\{ \begin{matrix} \frac{Ba_{w}}{\left( 1+\left( B-\omega\right)a_{w} \right)\left( 1-\omega a_{w} \right)} & \text{open sites} \\ 0 & \text{closed sites} \end{matrix} \right.$ | (S5) |
| --- | --- |

where $B$ and $\omega$ are mono- and multi-molecular equilibrium constants, respectively. Equation (S4) is similar to eq. (S5) in that (i) at low chemical potentials of water, monomolecular-like sorption occurs at discrete sites and (ii) sites that are inaccessible or “closed” have no water sorption. In eq. (S4), as the chemical potential of water increases, the strain increases enabling a greater sorption capacity at each accessible site, which is like the swelling GAB model in that multi-molecular sorption at discrete sites can occur. However, the swelling GAB model captures this phenomenon with multilayer equilibrium constants, while eq. (S4) uses a Langmuir isotherm to determine the fraction of accessible sites that are filled and those filled sites have multiple molecules sorbed ($n_{0}\left( 1+C\varepsilon_{v} \right))$.

In the poromechanics model, the bulk modulus, $K$, decays with increasing volumetric strain by eq. (S6):

| $K=K_{0}\exp(-bC\varepsilon_{v})$ | (S6) |
| --- | --- |

where $K_{0}$ is the bulk modulus of the dry state and $b$ is a parameter that describes the weakening of the material with increasing volumetric strain. Chen et al.^7^ assumed that the constant $C$ is equal to inverse the initial porosity $1/\phi_{0}$ under the supposition that volumetric strain results solely from porosity changes. Chen et al.^7^ derived an implicit equation for the volumetric strain as a function of the mechanical properties ($K_{0}, b$), the structural properties ($\phi_{0}, C$), sorption parameters ($B,n_{0}$), and environmental conditions ($a_{w},T$) during water sorption (eq. (S7)):

| $\varepsilon_{v}=-\frac{1}{b\phi_{0}C}\ln\{1-\left[ \alpha\left( \varepsilon_{v} \right)C\ln\left( 1+Ba_{w} \right)n_{0}\phi_{0}RT \right](b\phi_{0}C\exp\left( b\phi_{0} \right)/K_{0})\}$ | (S7) |
| --- | --- |

where $\alpha_{\mathrm{ads}}\left( \varepsilon_{v} \right)$ is the fraction of total hydrogen bonding sites available during sorption as a function of the volumetric strain (eq. (S8)):

| $\alpha_{\mathrm{ads}}\left( \varepsilon_{v} \right)=\int_{R_{c}/(1+\frac{C}{3}\varepsilon_{v})}^{\infty} f\left( R_{0} \right) dR_{0}$ | (S8) |
| --- | --- |

where $f\left( R_{0} \right)$ is the pore size distribution in the material, described as a Weibull distribution in the poromechanics model. As swelling occurs, the pore sizes increase, and only those pores with a radius larger than the critical radius $R_{c}$ are open. The integral of the pore-size distribution in eq. (S8) thus gives the fraction of sites that are open at a given strain $\varepsilon_{v}$. During desorption, it is assumed that all sites that were opened during swelling remain open until the material is completely dried, such that $\alpha_{\mathrm{des}}=\alpha_{\mathrm{ads}}\left( \varepsilon_{v,max} \right)$.

Equations (S7) and (S8) incorporate many mechanical properties of the material and captures non-linear strain versus site capacity relationships. It also inherently provides a maximum site capacity at the limit of high strains because $\alpha_{\mathrm{ads}}\leq1$. In contrast, the swelling GAB model we propose simplifies eqs. (S7) and (S8) by assuming that the strains are sufficiently small such that first-order Taylor series approximations describe (i) the equilibrium concentration of accessible sites as a linear function of volumetric strain and (ii) the volumetric strain is a linear function of temperature and the concentration of sorbed water, as given by eqs. (S9) and (S10):

| $\varepsilon_{v}=\alpha\left( T-T_{\mathrm{ref}} \right)+\beta c$ | (S9) |
| --- | --- |
| $L_{\mathrm{eq}}=L_{0}\left( T_{\mathrm{ref}} \right)+\gamma\varepsilon_{v}$ | (S10) |

Further, the site concentrations are described by a kinetic equation, allowing sites to close during desorption. This enables the swelling GAB model to capture sorption dynamics and complex sample histories such as varying initial and maximum relative humidities.

The max displacement form of the swelling GAB model used for fitting sorption isotherms gives a reasonable fit to the water sorption hysteresis observed on amorphous cellulose for the data reported by Chen et al.,^2^ as shown in Figure S3. The swelling GAB model also captures the volumetric strain, suggesting that the key difference between the poromechanics model and the swelling GAB model is how sorption at open sites occurs (eqs. (S4) and (S5)) and how site concentrations change with strain (eqs. (S8) and (S10)). Thus, we conclude that the swelling GAB model maintains the essence of the poromechanics model derived by Chen et al.^2^ while making a few model simplifications and also adding the ability to describe sorption dynamics and complex sample histories. The swelling GAB model is especially useful in the absence of simulations that can estimate pore-size distribution and mechanical properties of the material because the swelling GAB model instead relies on fitting first-order approximation parameters. In the poromechanics model fit to the experimental data, Chen et al.^7^ report values of (values in parentheses are in units reported by the original work) $n_{0}=0.098 g_{w} g_{p}^{-1}$ $(3.58 nm^{-3})$ and $B=153 \left( 0.043 Pa^{-1} \right)$, which are which are each within a factor of two of the similar parameters from the swelling GAB model, $n_{0}\approx L_{\mathrm{eq}}\left( \varepsilon_{v}=0 \right)+\phi=0.081 g_{w} g_{p}^{-1}$ and $B=82.2$, respectively, further illustrating the phenomenological similarities between the two models.


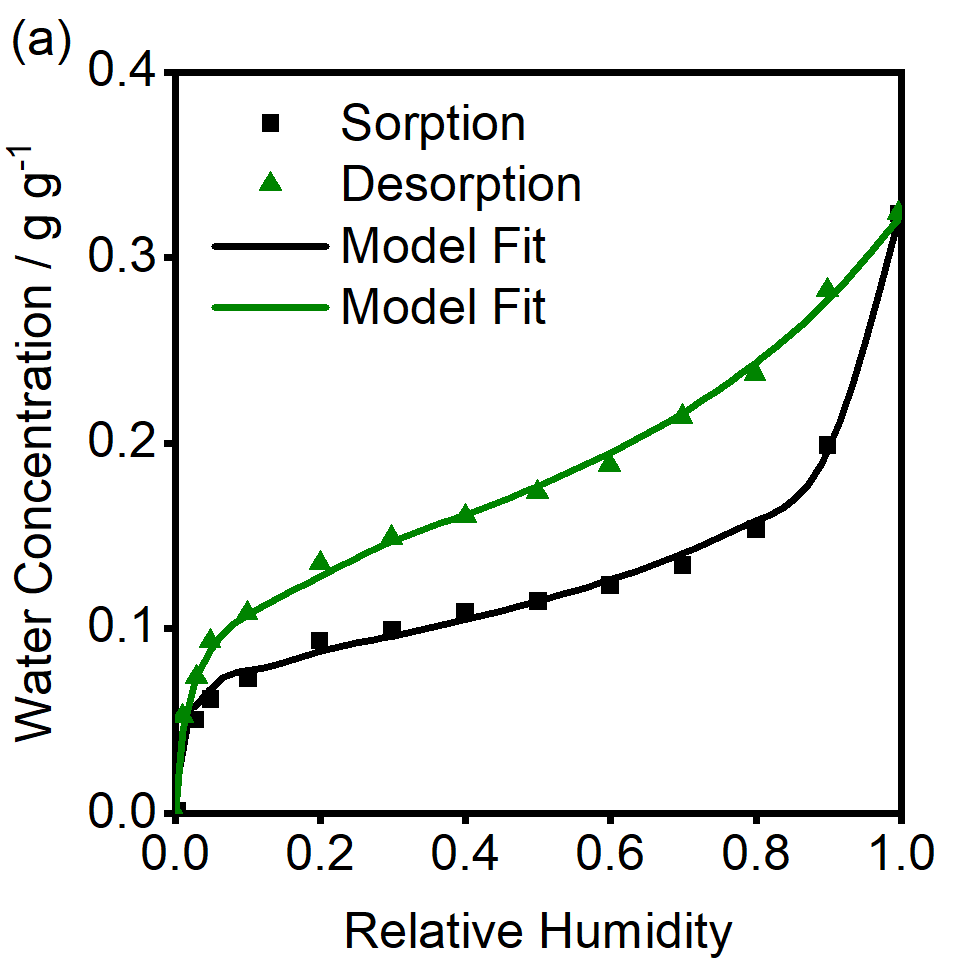

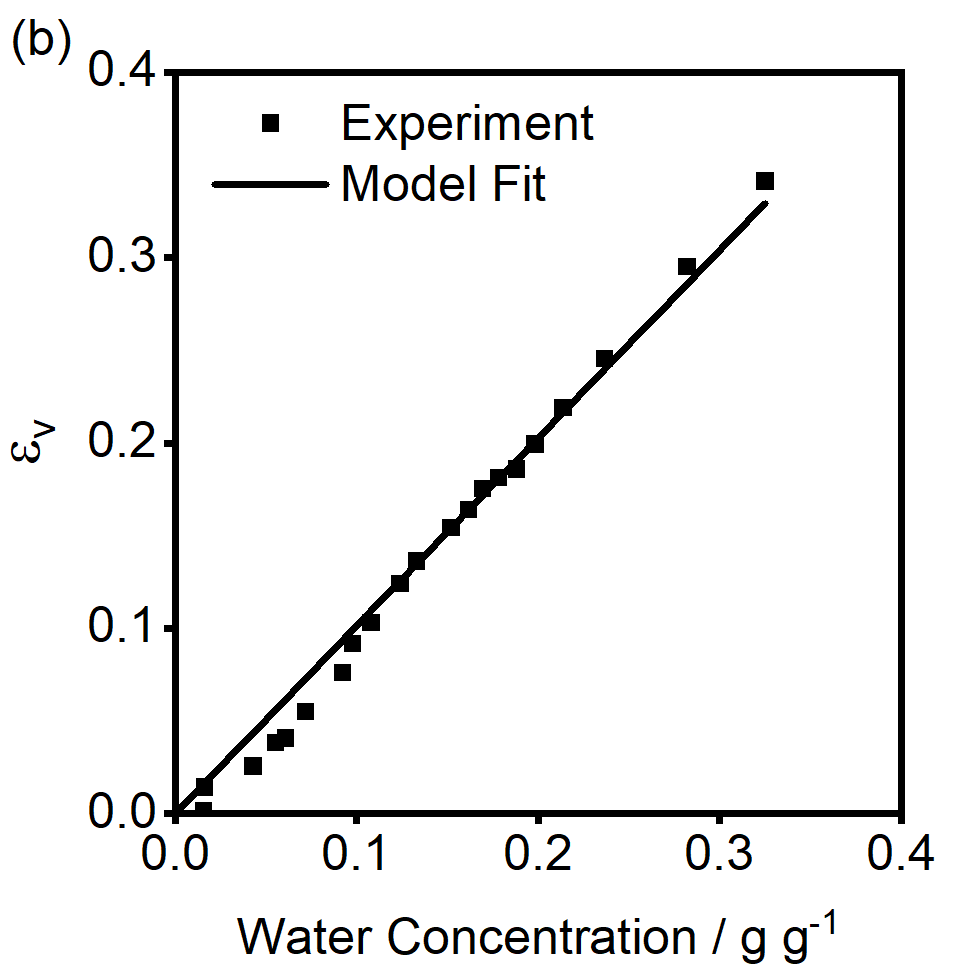


Figure S3. (a) Water concentration as a function of relative humidity on amorphous cellulose during sorption and desorption of water. (b) Volumetric strain as a function of sorbed water concentration during sorption and desorption. Data points are experimental data reported by Chen et al.^2^ and lines are model fits of the swelling GAB model using the max displacement method. with parameters $B=82.2$, $\omega=0.616$, $L_{\mathrm{eq}}\left( \varepsilon_{v}=0 \right)=0.056 g_{w} g_{p}^{-1}$, $\gamma=0.289 g_{w} g_{p}^{-1}$, $\beta=1.0138 g_{p} g_{w}^{-1}$, $\phi=0.0251 g_{w} g_{p}^{-1}$.

# S2. Thermo- and chemo-mechanical measurements of polyurethane

## Measurement of Glass Transition and Melting Point Temperatures

The glass transition and melting point temperatures of the glassy polyurethane sample were estimated by measuring the change in the linear strain as a function of temperature. The transition from the glassy to rubbery state leads to an increase in the thermal expansion coefficient.^1^ One method of estimating $T_{g}$ is by finding the intersection between the tangent lines of the strain versus temperature curve before and after the increase in thermal expansion.^1^ Using this technique, the $T_{g}$ for glassy polyurethane is estimated at ~413 K, as reported in Figure S1. At higher temperatures, the material begins to contract under the force of the pushrod. The temperature where this occurs is the melting point and is estimated here as ~487 K.^1^ The slope of the strain-temperature curve in the rubbery regime is decreased by the onset of the melting point, leading to a slight underestimate of the glass transition temperature.^1^ Regardless, the moisture sorption experiments in this study are at 298–333 K, in the regime where the polyurethane is in the glassy state.


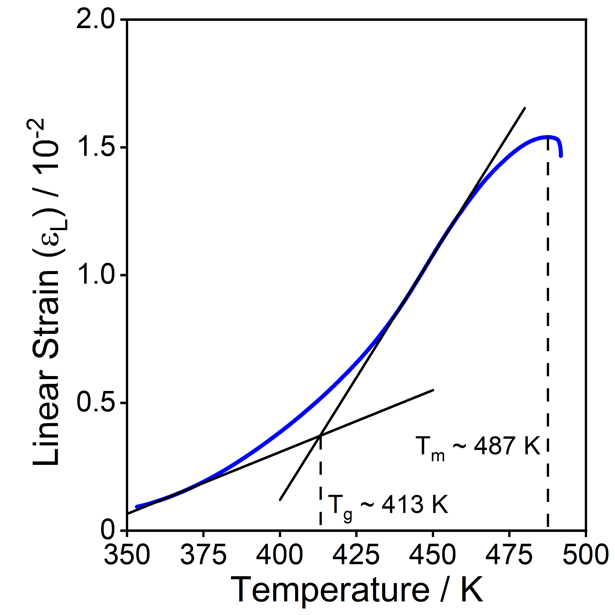


Figure S1. Linear strain as a function of temperature (0.033 K s^-1^ ramp rate; 0.200 N pushrod force; 0.33 cm^3^ s^-1^ dry N_2;_ RH = 0%). The increase in the thermal expansion coefficient (the slope of the strain-temperature curve) is attributed to the glassy-to-rubbery phase change. The intersection of the strain-temperature tangent lines is an estimate of the glass transition temperature, $T_{g}$ ~ 413 K. The melting temperature, $T_{m}$~ 487 K, is the temperature where the polyurethane softens, and the pushrod begins to compress the sample.

## Measurement of Thermal and Hygroscopic Expansion Coefficients

The thermal and sorption-induced swelling properties of the glassy polyurethane are reported in Figure S2. During sorption and desorption of moisture at 323 K, hysteresis is observed in the volumetric strain ($\varepsilon_{v}=\Delta V/V_{0}$) when compared as a function of the relative humidity (Figure S2a). However, when plotting the volumetric strain as a function of mass uptake per volume, the sorption and desorption curves overlay and are approximately linear, as shown in Figure S2b. Furthermore, the slope of this volumetric strain versus water uptake line is not a strong function of temperature, as demonstrated in Figure S2b, where $V_{0}=V(\text{RH}=0,T)$ for each temperature. The volume change per mass of sorbed water in glassy polyurethane is 1.82 cm^3^ g_w_^-1^, as determined by the slope of the best-fit line to the data in Figure S2b. This change in volume is greater than the specific volume of water (~ 1 cm^3^ g_w_^-1^), suggesting that mixing water with glassy polyurethane is a non-ideal process with a positive excess volume of mixing.

The collapse of the volumetric strain curve when plotting against mass uptake is also observed on amorphous cellulose, which exhibits similar sorption hysteresis to glassy polyurethane.^2^ In amorphous cellulose, the density of Langmuir-like sites are a function of the volumetric strain. We report the volumetric strain of glassy polyurethane as a function of mass uptake (Figure S2b) and temperature (Figure S2c), from which we determine the hygroscopic expansion coefficient, $\beta=d\varepsilon_{v}\text{/}d\left( m_{w}\text{/}m_{p} \right)=0.62 g_{p} g_{w}^{-1}$, and the thermal expansion coefficient, $\alpha=d\varepsilon_{v}\text{/}dT=1.52\times{10}^{-4} K^{-1}$, respectively.


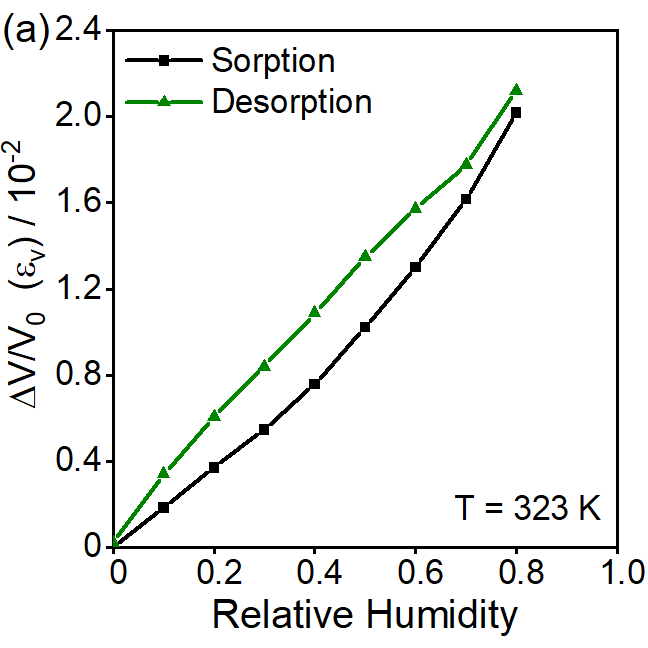

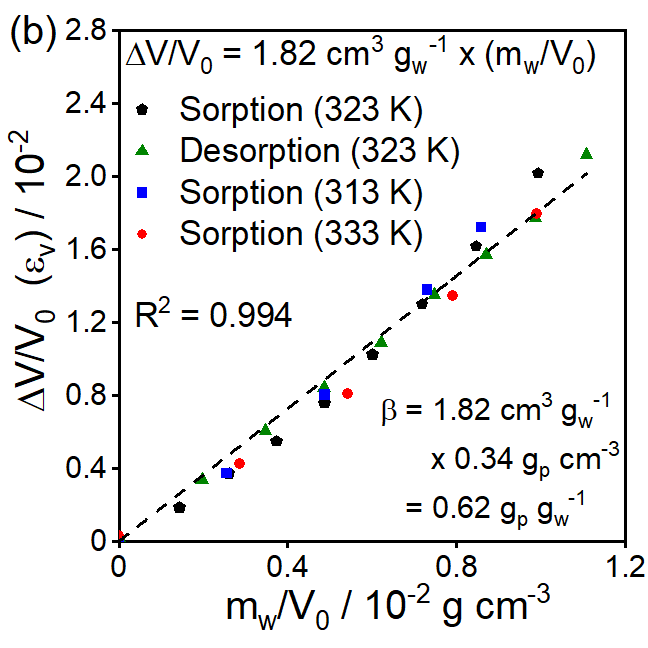

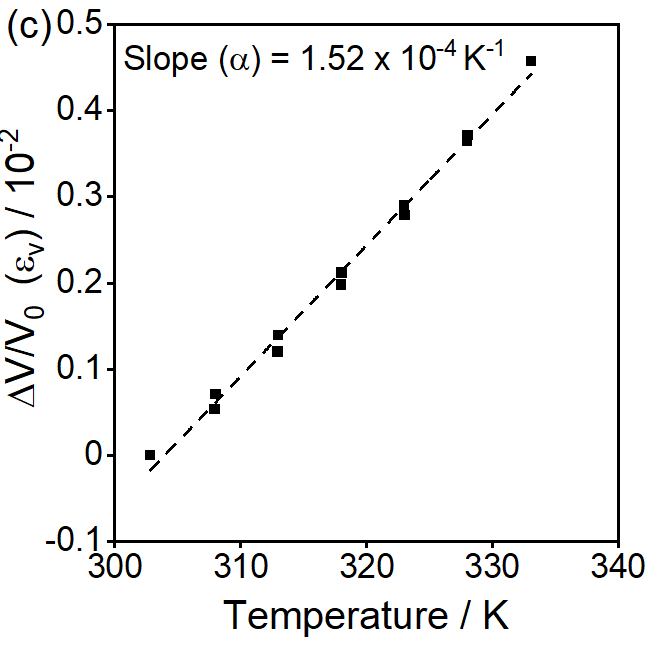


Figure S2. (a) Relative change in equilibrium volume as a function of relative humidity at T = 323 K during sorption and desorption of water. (b) Relative volume change as a function of mass uptake of water per volume at 313–333 K. Sorption and desorption data at 323 K overlay. The slope of the data (1.82 cm^3^ g^-1^) multiplied by the polymer density (0.34 g cm^-3^) gives the hygroscopic expansion coefficient (0.62 g_p_ g_w_^-1^). (c) Equilibrium relative volume change of dry polymer as a function of temperature. Volumetric data during heating and cooling overlay. The slope of this data gives the thermal expansion coefficient (1.52 × 10^-4^ K^‑1^).

# S3. Discussion on estimating *k* and *n* for data fit in Figure 4 by analysis of eigenvalues and eigenvectors of the Hessian Matrix

The eigenvalues and eigenvectors of the Hessian matrix provide a quantitative comparison of the confidence of parameter estimates.^8^ The Hessian matrix, $H$, was approximated from the Jacobian, $J$, by the equation $H\approx J^{'}J$, where the Jacobian is given by eq. (S11):

| $J=\left[ \begin{matrix} \frac{\partial\hat{y}_{1}}{\partial x_{1}} & \cdots& \frac{\partial\hat{y}_{1}}{\partial x_{m}} \\ \vdots& \ddots& \vdots\\ \frac{\partial\hat{y}_{n}}{\partial x_{1}} & \cdots& \frac{\partial\hat{y}_{n}}{\partial x_{m}} \end{matrix} \right]$ | (S11) |
| --- | --- |

where $\hat{y}_{n}$ are the model predicted data points and $x_{m}$ are the parameters. The smallest eigenvalues of the Hessian matrix are associated with the eigenvector of parameters that are least well estimated from the fit to the experimental data. The eigenvalues of the Hessian matrix are reported in Figure S4 labeled with the most significant components of the associated eigenvectors. Figure S4 shows that the parameters $k$ and $n$ are the least well estimated from the experimental data and that they are correlated to each other. This is illustrated in Figure S5 which compares the best fit curves for $n=1.0, n=4.7$, and $n=12.9$. The fits for $n=4.7$ and $n=12.9$ are similar and demonstrate the weak estimation of $k$ and $n$ by the experimental data. However, $n$ cannot be as small as 1.0 and still obtain a good fit, indicating the weakness of the fit still constrains $n$ to be sufficiently large to model the experimental data. Thus, the kinetic equation for describing site concentration dynamics greatly improves the dynamic fit, it is just that estimates for the kinetic parameters are imprecise. Better estimates for $k$ and $n$ could be obtained by measuring sorption dynamics on much longer timescales.


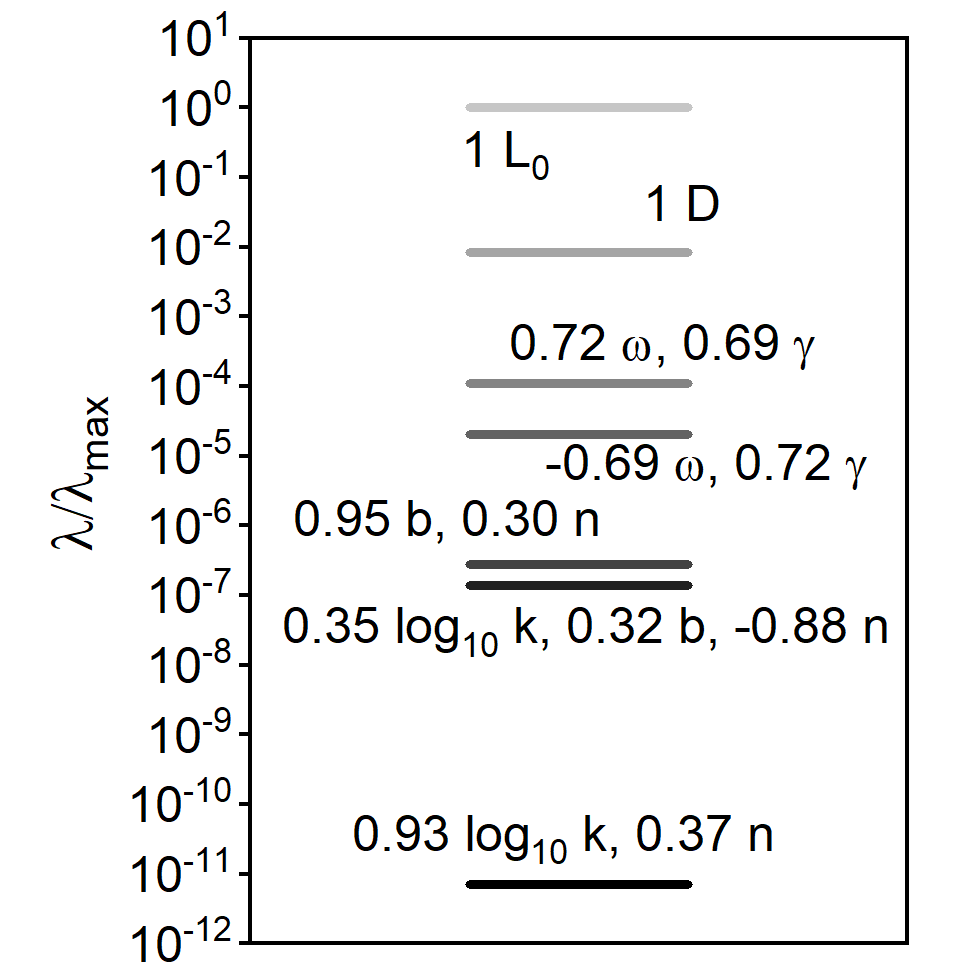


Figure S4. Eigenvalues and major components of the associated eigenvectors of the Hessian matrix for the parameters estimated from the dynamic data in Figure 4.


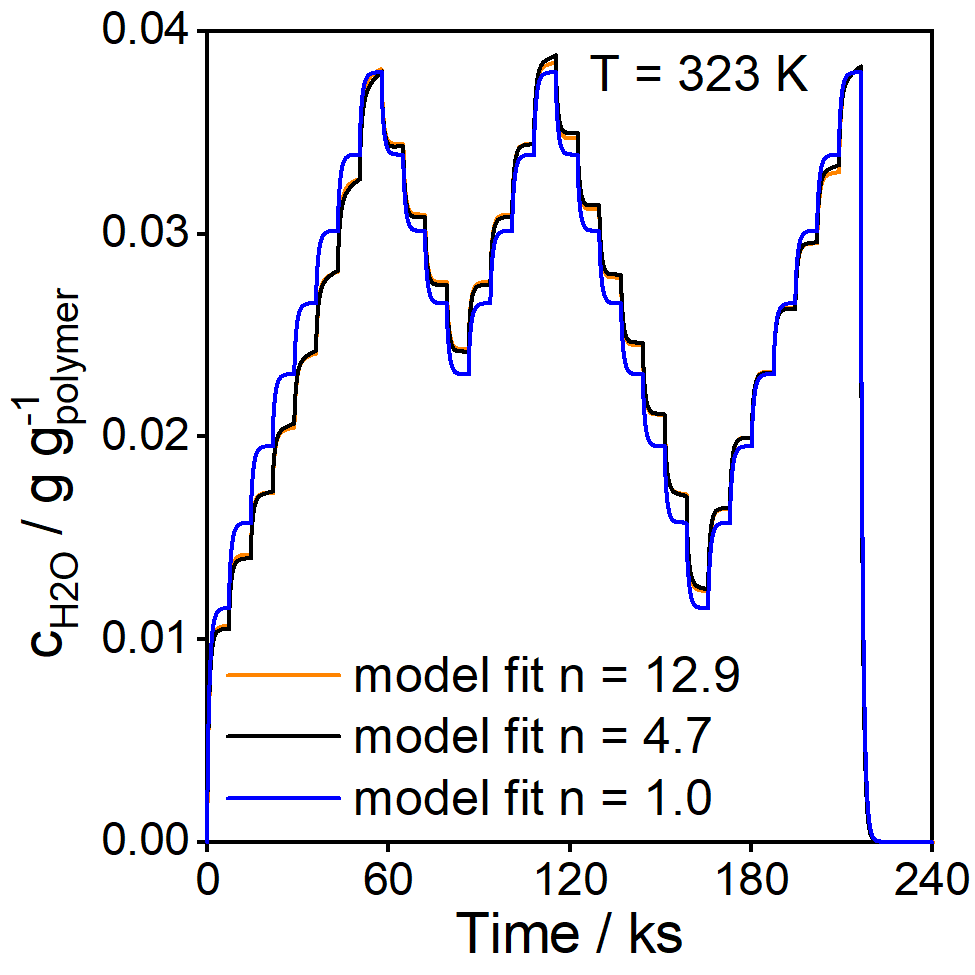


Figure S5. Comparison of model fits for $n=12.9 \left( k=3.61 \times{10}^{27} s^{-1} \right)$, $n=4.7 (k=7.9 \times{10}^{6} s^{-1})$, and $n=1.0 (k=99.4 s^{-1})$. The $n=4.7$ and $n=12.9$ fits are very similar with only slight deviations in isotherm points and dynamics, but the $n=1.0$ gives a significantly worse best-fit isotherm.

# References

(1) *Thermal Analysis of Polymers: Fundamentals and Applications*; Menczel, J. D., Prime, R. B., Eds.; Wiley: Hoboken, NJ, 2009.

(2) Chen, M.; Coasne, B.; Guyer, R.; Derome, D.; Carmeliet, J. Role of Hydrogen Bonding in Hysteresis Observed in Sorption-Induced Swelling of Soft Nanoporous Polymers. *Nat Commun* **2018**, *9* (1), 3507. https://doi.org/10.1038/s41467-018-05897-9.

(3) Vrentas, J. S.; Vrentas, C. M. Hysteresis Effects for Sorption in Glassy Polymers. *Macromolecules* **1996**, *29* (12), 4391–4396. https://doi.org/10.1021/ma950969l.

(4) Doghieri, F.; Sarti, G. C. Nonequilibrium Lattice Fluids: A Predictive Model for the Solubility in Glassy Polymers. *Macromolecules* **1996**, *29* (24), 7885–7896. https://doi.org/10.1021/ma951366c.

(5) Minelli, M.; Sarti, G. C. 110th Anniversary: Gas and Vapor Sorption in Glassy Polymeric Membranes—Critical Review of Different Physical and Mathematical Models. *Ind. Eng. Chem. Res.* **2020**, *59* (1), 341–365. https://doi.org/10.1021/acs.iecr.9b05453.

(6) Vrentas, J. S.; Duda, J. L.; Ling, H. C. Antiplasticization and Volumetric Behavior in Glassy Polymers. *Macromolecules* **1988**, *21* (5), 1470–1475. https://doi.org/10.1021/ma00183a042.

(7) Chen, M.; Coasne, B.; Guyer, R.; Derome, D.; Carmeliet, J. A Poromechanical Model for Sorption Hysteresis in Nanoporous Polymers. *J. Phys. Chem. B* **2020**, *124* (39), 8690–8703. https://doi.org/10.1021/acs.jpcb.0c04477.

(8) Gutenkunst, R., N.; Waterfall, J. J.; Casey, F. P.; Brown, K. S.; Myers, C. R.; Sethna, J. P. Universally Sloppy Parameter Sensitivities in Systems Biology Models. *PLoS Comput Biol* **2007**, *3* (10). https://doi.org/e189. doi:10.1371/journal.pcbi.0030189.
